# Supplementary material for: Prediction of strong Cu(I)–He interaction at open metal sites enables isotope-selective helium adsorption
Source: Nat Commun. 2026 Mar 26;17:2952. doi: 10.1038/s41467-026-70901-6 (PMC13031845; doi:10.1038/s41467-026-70901-6)
Supplement: Supplementary file 1 — Supplementary Information [file 41467_2026_70901_MOESM1_ESM.pdf]

Supplementary Information

Prediction of strong Cu(I)–He interaction at open metal sites enables isotope-selective helium adsorption

Elvira Gouatieu Dongmo, Shubhajit Das, Felix Moncada, Toshiki Riemer-Wulf, Thomas Heine,*

*Correspondence author: *thomas.heine@tu-dresden.de*

Table of Contents

1. Supplementary Note 1. Accurate Modeling of Cu–He Interactions in Cu(I) complexes: CCSD(T) and DLPNO-CCSD(T) PES Scans, Morse potential and Charge-Induced-Dipole potential 5

1.1. QTAIM Analysis of $Cu^{+}(X^{-})He$ Complexes with X=F^{-}, HO^{-}, HS^{-}, Cl^{-} and Br^{-} 7

1.2. QTAIM Analysis of the water dimer, Argon dimer and dihydrogen molecule. 8

1.3. EDA-NOCV analysis of the Cu(I)-He bond 10

2. Supplementary Note 2. Finite difference method (FDM) applied to Schrödinger equation describing the Cu-He bond 13

2.1. Quartic anharmonic correction 13

3. Supplementary Note 3. Detailed Calculations of adsorbed He Isotope Separation on Strongly Attractive Adsorption Site 20

4. Supplementary Note 4. Vibrational mode decomposition analysis of Cu(I)/UiO-66 32

5. Supplementary Note 5. DVR zero-point energy comparisons 34

5.1. DVR assessment of the separability of the He modes 35

5.2. Cu-He Stretch partition function validation 36

5.3. He perpendicular modes partition function validation 39

6. Fitted potential parameters 41

6.1. Small complexes 41

6.2. Cluster models of molecules and materials 45

Supplementary references 47

List of Supplementary Figures

Supplementary Fig. 1. Born–Oppenheimer dissociation energy for all Cu(I) complexes. 6

Supplementary Fig. 2. Structures of the $Cu^{+}(X^{-})He$ complexes with X=F^{-}, HO^{-}, HS^{-}, Cl^{-} and Br^{-} results of the QTAIM analysis. 7

Supplementary Fig. 3. QTAIM analysis of the water dimer, Argon dimer and dihydrogene molecule. 8

Supplementary Fig. 4. Potential energy values along x direction. 19

Supplementary Fig. 5. Optimized structure of the uncharged cluster containing the benzotriazolate (bta^-) ligand of formula $Cu(I)Zn_4Cl(bta)_6$ (Model-I) and its smaller truncated version (Model-II) at PBE0-D3/def2-TZVP level......26

Supplementary Fig. 6. Adsorbed He atom on three SBUs from the HEALED-library. 29

Supplementary Fig. 7. Decomposition of the vibrational mode at 132.3 cm^{-1} for the Cu(I)/UiO-66 cluster 33

Supplementary Fig. 8. Schematic depiction of the He positions (in light blue) used to generate the Cu^+H_2O-He 3D-DVR grid. 34

Supplementary Fig. 9. Morse plus charge induced potential energies, V(R), and 1D-DVR energies, E_{vib}, for CuF-$^{3/4}$He (left) and $Cu[(Cu_4Cl)(ttmp)_2]_2$ -$^{3/4}$He (right)...37

Supplementary Fig. 10. 1D-DVR partition function analysis of $^{3/4}$He in CuF system and $Cu[(Cu_4Cl)(ttmp)_2]_2$ cluster. 38

Supplementary Fig. 11. 1D-DVR bound vibrational states for the quartic potential of the He-6c2 complex. 39

Supplementary Fig. 12. 1D-DVR partition function analysis of $^{3/4}$He in 6c2 cluster: Comparison of q_{full} and q_{approx} partition functions and their ratios..40

List of Supplementary Tables

Supplementary Table 1. QTAIM analysis for small Cu(I) complexes. 9

Supplementary Table 2. Nature of Cu(I)–He Bonding: EDA-NOCV analysis of Cu(I)-He bonding in small complexes. 11

Supplementary Table 3.Vibrational analysis for Cu(I)–He complexes at the MP2/def2-TZVPP level.......................15

Supplementary Table 4. Summary of computed zero-point energies for adsorbed He isotopes on Cu(I) complexes..17

Supplementary Table 5. Predicted separation factors ($^4He/^3He$) for adsorbed He isotopes in all Cu(I)-complexes at low temperatures..23

Supplementary Table 6. Influence of threshold settings on interaction parameters..24

Supplementary Table 7. Summary of QTAIM descriptors for adsorbed He on various material models. 25

Supplementary Table 8. Comparison of adsorption energies using DFT and high-level methods. 27

Supplementary Table 9. Adsorbed He isotopes on various cluster models...28

Supplementary Table 10. Structural and energetic properties of dsorbed He isotopes on various cluster models.......30

Supplementary Table 11. Predicted separation factors for adsorbed He isotopes on various cluster models of molecules and materials calculated at DLPNO-CCSD(T)/cc-pVTZ(aug-cc-pVTZ for Cu, He) level of theory. 31

Supplementary Table 12. Comparison between 3D-DVR and FDM plus quartic potential zero-point energies (in kJ/mol) 35

Supplementary Table 13. Comparison between 1D-DVR and FDM ZPEs for the Cu-He stretch and between 2D-DVR and quartic potential ZPEs for the two perpendicular He modes. 36

Supplementary Table 14. Morse and charge-induced dipole potential parameters with MP2 method. 41

Supplementary Table 15. Quartic potential parameters for orthogonal modes for small complexes. 42

Supplementary Table 16. Morse and charge-induced dipole potential parameters with CCSD(T) and DLPNO-CCSD(T) methods for small complexes. 43

Supplementary Table 17. Quartic potential parameters for orthogonal modes for small complexes. 44

Supplementary Table 18. Morse and charge-induced dipole potential parameters for cluster models of molecules and materials. Fitted Morse and charge-induced dipole potential parameters describing the stretching mode potential energy curves of Cu(I) complexes computed at DLPNO-CCSD(T)/ cc-pVTZ(aug-cc-pVTZ for Cu, He level of theory.......45

Supplementary Table 19. Quartic potential parameters for orthogonal modes for cluster models of molecules and materials. Parameters of quartic potential energy curves for the orthogonal modes of Cu(I) complexes, computed at the DLPNO-CCSD(T)/ cc-pVTZ(aug-cc-pVTZ for Cu, He) level of theory. 46

1. Supplementary Note 1. Accurate Modeling of Cu–He Interactions in Cu(I) complexes: CCSD(T) and DLPNO-CCSD(T) PES Scans, Morse potential and Charge-Induced-Dipole potential

We represented the Cu^{+}–He interaction with a potential composed of a Morse potential augmented with a charge-induced dipole potential that is switched off for short distances using a Fermi switching function

$$V_{MCID}(z) = V_{Morse}(z) + (1 - F_{fermi}(z)) * V_{CID}(z)$$

with the components

$$V_{Morse}(z_e) = D_e \left[\left(1 - \exp\left(-a(z - z_e)\right)\right)^2 - 1 \right]$$

$$F_{fermi}(z) = \frac{1}{1 + e^{\left(\frac{z - z_c}{k_B T}\right)}}$$

$$V_{CID}(z) = -Cz^{-4}$$

The parameters of the potentials, namely

- D_e: the well depth or the dissociation energy in kJ mol^{-1}
- z_e: the equilibrium distance in Å
- a: the stiffness parameter in $Å^{-1}$
- C: the charge-induced charge parameter in $Å^4$kJ mol^{-1}

have been obtained using CCSD(T)/aug-cc-pVTZ carried out in the Gaussian 16 program package(*1*) and, for validation, using DLPNO-CCSD(T)/cc-pVTZ (aug-cc-pVTZ for Cu, He) calculations performed in ORCA 5.0.1.(*2*) The parameters of the switching function have been set to

- $z_c = 1.5 z_e$
- $k_B T$= 2.2 · ZPE

During fitting, data points near the minimum were weighted very heavily (weight = 10 000) to ensure an accurate description of the minimum, while points at both very short and long range carried much smaller weights, preventing overfitting in regions that matter less physically. Figure S1 illustrates the fitted potential curve obtained using the described MCID approach. The fitted parameters for the systems studied in the manuscript are presented in Suppl. Tables 14, 16 and 18.

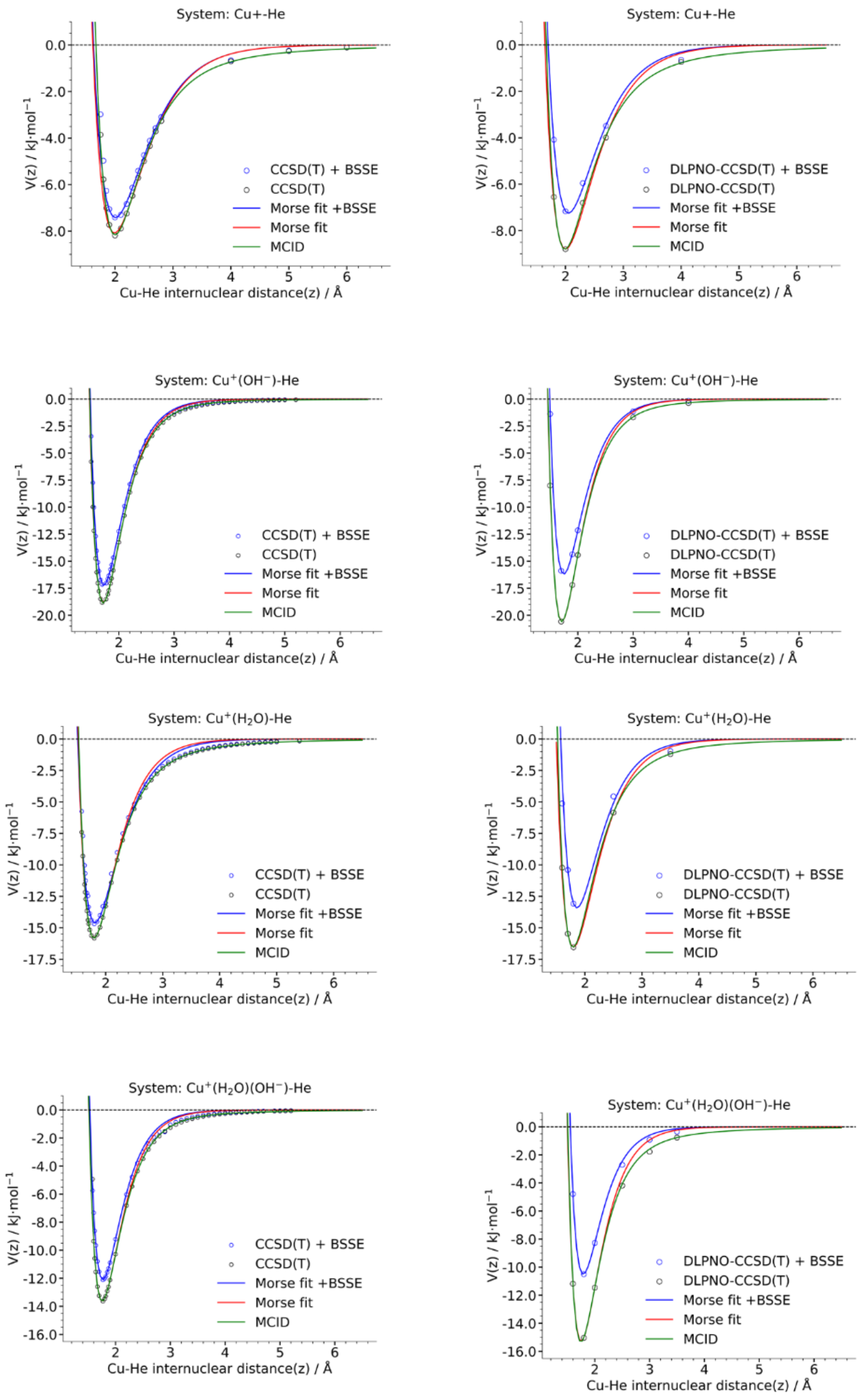

Supplementary Fig. 1. Born–Oppenheimer dissociation energy for all Cu(I) complexes. Potential energy curves for four Cu(I) complexes computed at the CCSD(T)/aug-cc-pVTZ (left side) and DLPNO-CCSD(T)/cc-pVTZ (aug-cc-pVTZ for Cu, He) (right side) levels. Blue and black circles mark the data points. The red line is a Morse-potential fit while the blue line shows a Morse potential with BSSE-corrected energies. The green curve shows V_{MCID} as described above.

1.1. QTAIM Analysis of $Cu^{+}(X^{-})He$ Complexes with X=F^{-}, HO^{-}, HS^{-}, Cl^{-} and Br^{-}

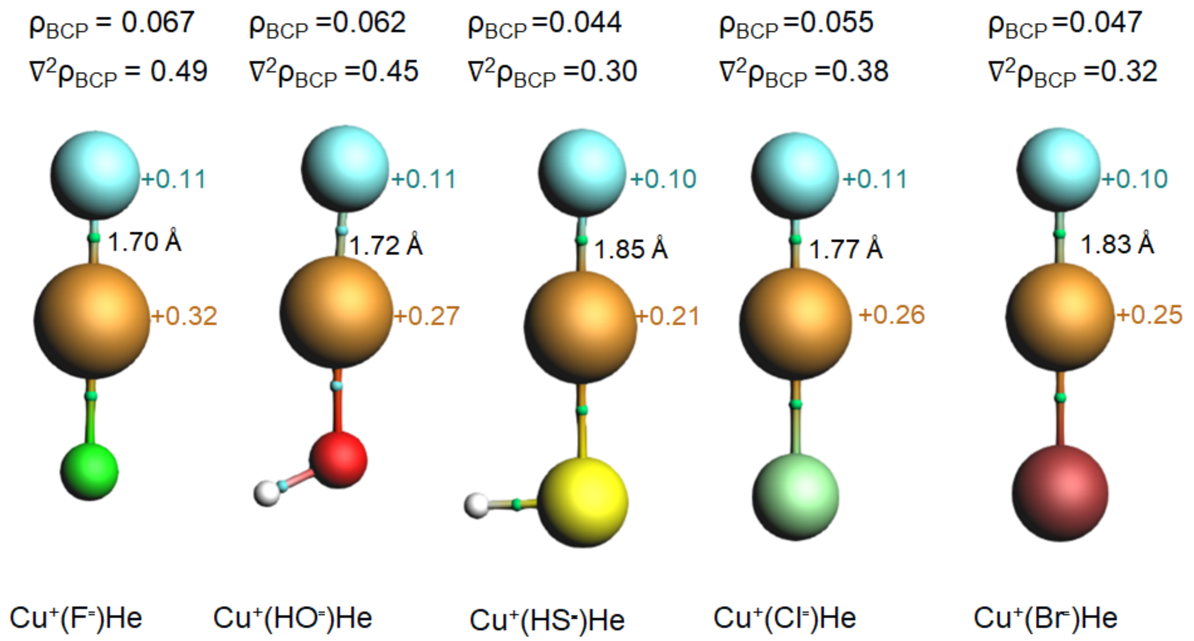

Supplementary Fig. 2. Structures of the $Cu^{+}(X^{-})He$ complexes with X=F^{-}, HO^{-}, HS^{-}, Cl^{-} and Br^{-} results of the QTAIM analysis. Cu-He bond lengths (CCSD(T)/aug-cc-pVTZ level) are shown next to the bond paths and critical points as obtained by the QTAIM analysis, while the Hirshfeld charges are shown for Cu (in brown) and He (in light blue). Values of the electron density ρ_{BCP} and its Laplacian $\nabla^2\rho_{BCP}$ are reported in atomic units (a.u.).

1.2. QTAIM Analysis of the water dimer, Argon dimer and dihydrogen molecule.

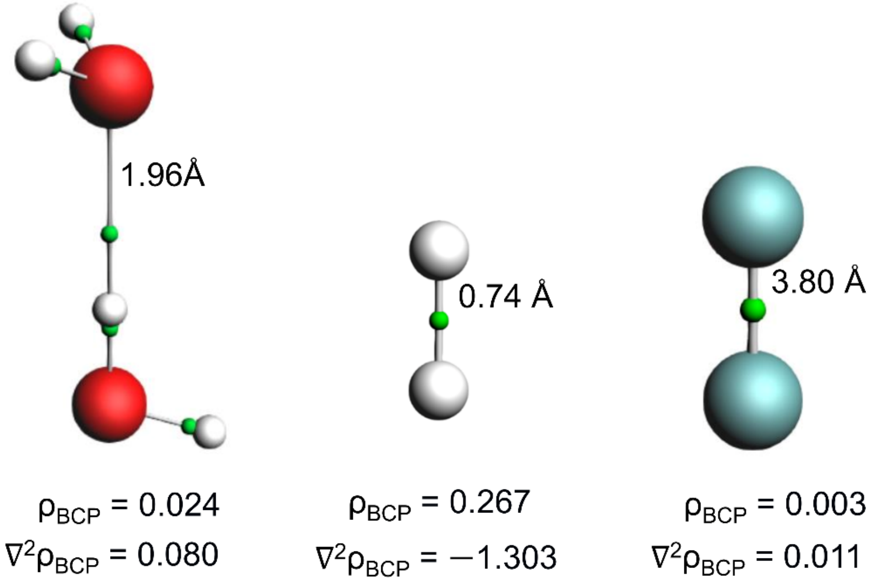

Supplementary Fig. 3. QTAIM analysis of the water dimer, Argon dimer and dihydrogene molecule. Bond lengths at the CCSD(T)/aug-cc-pVTZ level are indicated. Colour scheme: O−red, H−white, Ar−cyan-green. Bond critical points are shown as small green spheres. Values of the electron density ρ_{BCP} and its Laplacian $\nabla^2\rho_{BCP}$ are reported in atomic units (a.u.).

Supplementary Table 1. QTAIM analysis for small Cu(I) complexes.
Local QTAIM properties calculated at the PBE0-D3(BJ)/TZ2P level of theory on CCSD(T)/aug-cc-pVTZ geometries (for validation purposes at DLPNO-CCSD(T)/ cc-pVTZ(aug-cc-pVTZ for Cu, He) geometries, values given in the square brackets). Hirshfeld charges of the model systems are given for Cu in the bare system q_{Cu}, in the systems with adsorbed helium q_{CuHe} and their differences $\Delta_{ads}q_{Cu}$, as well as for the adsorbed He atoms q_{He}, electron density ρ_{BCP} and its Laplacian $\nabla^2{}_{BCP}$ at the BCP between Cu^+ and He and the distance of the BCP to the copper z_{Cu-BCP} and helium z_{He-BCP} centers.

Systems	q_{Cu} (e)	q_{CuHe} (e)	$\Delta_{ads}q_{Cu}$ (e)	q_{He} (e)	ρ_{BCP} ($e \cdot r_{Bohr}$)	$\nabla^2{}_{BCP}$ ($e \cdot r_{Bohr}$)	$z_{Cu\text{–}BCP}$ (Å)	$z_{He\text{–}BCP}$ (Å)
$Cu^+(F^-)He$	+0.423<br>[+0.423]	+0.319<br>[0.332]	−0.104<br>[−0.091]	+0.112<br>[0.096]	0.067<br>[0.036]	0.489<br>[0.258]	0.97<br>[1.063]	0.73<br>[0.837]
$Cu^+(H_2O)(OH^-)He$	+0.370<br>[+0.370]	+0.284<br>[0.278]	−0.086<br>[−0.092]	+0.100<br>[+0.104]	0.055<br>[0.059]	0.383<br>[0.423]	1.01<br>[0.99]	0.76<br>[0.75]
$Cu^+(Cl^-)He$	+0.360<br>[+0.360]	+0.264<br>[0.259]	−0.096<br>[−0.101]	+0.105<br>[+0.111]	0.055<br>[0.068]	0.380<br>[0.479]	1.01<br>[0.97]	0.76<br>[0.73]
$Cu^+(HO^-)He$	+0.355<br>[+0.355]	+0.265<br>[+0.264]	−0.090<br>[−0.091]	+0.106<br>[+0.108]	0.062<br>[0.066]	0.454<br>[0.484]	0.98<br>[0.97]	0.74<br>[0.73]
$Cu^+(Br^-)He$	+0.334<br>[+0.334]	+0.247<br>[+0.252]	−0.087<br>[−0.082]	+0.099<br>[+0.094]	0.047<br>[0.038]	0.317<br>[0.253]	1.04<br>[1.07]	0.79<br>[0.82]
$Cu^+(HS^-)He$	+0.284<br>[+0.284]	+0.211<br>[+0.213]	−0.073<br>[−0.071]	+0.095<br>[+0.091]	0.044<br>[0.038]	0.296<br>[+0.25]	1.05<br>[+1.07]	0.80<br>[+0.83]
Cu^+	+1.000<br>[+1.000]	+0.900<br>[+0.897]	−0.100<br>[−0.103]	+0.101<br>[+0.103]	0.032<br>[0.034]	0.184<br>[0.195]	1.13<br>[1.12]	0.87<br>[0.86]
$Cu^+(H_2O)He$	+0.721<br>[+0.721]	+0.599<br>[+0.597]	−0.122<br>[−0.124]	+0.120<br>[+0.122]	0.050<br>[0.052]	0.325<br>[0.341]	1.03<br>[1.02]	0.79<br>[0.78]

1.3. EDA-NOCV analysis of the Cu(I)-He bond

The nature of the bond between Cu(I) and He has been analyzed by energy decomposition analysis (EDA) (3, 4) coupled with natural orbital for chemical valence (NOCV) (5–7) method using the ADF 2025.105 program package (8). EDA-NOCV calculations were carried out using the hybrid PBE0 functional with D3 dispersion correction and Becke-Johnson damping (BJ), termed PBE0-D3(BJ), together with the Slater-type TZ2P basis set (9). The geometries used were optimized at the CCSD(T)/aug-cc-pVTZ and DLPNO-CCSD(T)/cc-pVTZ (Cu, He: aug-cc-pVTZ) levels. The EDA-NOCV method allows the decomposition of the total interaction energy (ΔE_{int}) between two fragments into four physical components as follows (10, 11):

$$\Delta E_{int} = \Delta E_{elstat} + \Delta E_{pauli} + \Delta E_{orb} + \Delta E_{int}$$

Where the terms include the quasi-electrostatic contribution, ΔE_{elstat}, the repulsive interaction energy, ΔE_{pauli}, the orbital interaction energy ΔE_{orb}, and the dispersion interaction ΔE_{disp}.

Supplementary Table 2. Nature of Cu(I)–He Bonding: EDA-NOCV analysis of Cu(I)-He bonding in small complexes. EDA-NOCV results for He binding to small Cu(I) complexes calculated at the PBE0-D3(BJ)/TZ2P level of theory on CCSD(T)/aug-cc-pVTZ geometries and DLPNO-CCSD(T)/ cc-pVTZ(aug-cc-pVTZ for Cu, He) geometries (values given in the square brackets). All energies in kJ·mol⁻¹, bond distances in Å.

Systems	d_{CuHe}	E_{ads}^{B0} [a]	ΔE_{int}	ΔE_{disp}	ΔE_{pauli}	ΔE_{elstat}	ΔE_{orb}	ΔE_1 (σ(He) → Cu(d))[b]	ΔE_2 (Cu(d) → σ*(He))[c]	$\Delta E_1 + \Delta E_2$
$Cu^+(F^-)$	1.70	−19.4	−21	−1	49	−33 (48%)	−36 (52%)	−16 (48%)	−17 (52%)	−33
	[1.72]	[−18.6]	[−19]	[−1]	[16]	[−15 (44%)]	[−19 (56%)]	[−13 (72%)]	[−5 (28%)]	[−18]
$Cu^+(OH^-)$	1.72	−17.1	−18	−1	47	−31 (48%)	−33 (52%)	−19 (66%)	−10 (34%)	−29
	[1.70]	[−16.0]	[−17]	[−1]	[52]	[−33 (49%)]	[−35 (51%)]	[−20 (65%)]	[−11 (35%)]	[−31]
$Cu^+(OH^-)(H_2O)$	1.77	−12.1	−15	−1	46	−29 (48%)	−31 (52%)	−13 (46%)	−15 (54%)	−28
	[1.80]	[−11.0]	[−16]	[−1]	[36]	[−24 (47%)]	[−27 (53%)]	[−10 (42%)]	[−14 (58%)]	[−24]
$Cu^+(Cl^-)$	1.77	−12.1	−14	−1	44	−27 (47%)	−30 (53%)	−12 (43%)	−16 (57%)	−28
	[1.80]	[−10.9]	[−13]	[−1]	[62]	[−37 (50%)]	[−37 (50%)]	[−14 (%)]	[−20 (%)]	[−34]
$Cu^+(Br^-)$	1.83	−9.7	−12	−1	38	−23 (47%)	−26 (53%)	−10 (42%)	−14 (58%)	−24
	[1.81]	[−8.6]	[−12]	[−1]	[27]	[−17 (45%)]	[−21 (55%)]	[−10 (50%)]	[−10 (50%)]	[−20]
$Cu^+(HS^-)$	1.85	−7.5	−9	−1	38	− 22 (48%)	−24 (52%)	− 11 (48%)	−12 (52%)	−23
	[1.87]	[−6.6]	[−9]	[−1]	[31]	[−18 (46%)]	[−21 (54%)]	[−10 (56%)]	[−8 (44%)]	[−18]
	1.82	−14.4	−21	−1	36	−22 (39%)	−34 (61%)	−18 (55%)	−15 (45%)	−33

$Cu^+(H_2O)He$	[1.80]	[−13.1]	[−21]	[−1]	[39]	[−24 (41%)]	[−35 (59%)]	[−18 (53%)]	[−16 (47%)]	[−34]
Cu^+	2.0<br>[2.0]	−7.6<br>[−7.2]	−13<br>[−14]	−1<br>[−1]	36<br>[23]	−19 (39%)<br>[−13 (35%)]	−30 (61%)<br>[−24 (65%)]	−15 (50%)<br>[−14 (61%)]	−15 (50%)<br>[−9 (39%)]	−30<br>[−23]

All fragments are closed-shell species. $^{a}E_{ads}^{B0}$ was calculated at the CCSD(T)/aug-cc-pVTZ and at the DLPNO-CCSD(T)/ cc-pVTZ(aug-cc-pVTZ for Cu, He) levels of theory with BSSE-corrected. [b]Percentage values give the relative contributions to the partitioned energy decomposition attractive analysis (pEDA) terms ΔE_{elstat} and ΔE_{orb}. [c]Percentage values give the relative contributions of the NOCV to ΔE_{orb}.

2. Supplementary Note 2. Finite difference method (FDM) applied to Schrödinger equation describing the Cu-He bond

The FDM is used to solve the one-dimensional time-independent Schrödinger equation (1D-TISE)

$$\left[-\frac{\hbar^2}{2\mu}\left(\frac{\partial^2}{\partial z^2}\right)+V_{MCID}(z)\right]\psi(z)=E\psi(z)$$

of the Cu^+–He bond described by the parameterized $V_{MCID}(z)$ potential for model complexes and material models. The Crank–Nicolson scheme(*12*) was employed to evaluate the differential operator at each grid-point for second-order accuracy. Hence, by discretizing the domain into $N+2$ grid points, the approximation used to express the second derivative along the z-direction ($D=\frac{\partial^2}{\partial z^2}$) is the three-point stencil represented by a tridiagonal matrix:

$$D=\begin{pmatrix} -2 & 1 & 0 & 0 & \cdots & 0 \\ 1 & -2 & 1 & 0 & \dots & 0 \\ 0 & 1 & -2 & 1 & \dots & 0 \\ \vdots & \ddots\ 0 & 1 & \ddots & \ddots & \vdots \\ 0 & 0\ \ 0 & & \vdots & \ddots & 1 \\ 0 & 0\ \ 0 & & \dots & 1 & -2 \end{pmatrix}_{N\times N}$$

Uniform grid spacing with a step size of $h=\Delta z=0.001\text{Å}$ and a truncation error of $O(h^2)$ was chosen. The next step involves the construction of the differentiation matrices and the numerical formulation of the potential energy (V_{MCID}). This potential energy is converted into a matrix of size N^2, with the main diagonal being occupied by the N elements. After the construction of the nuclear Hamiltonian, one can obtain the normalized ground state wave function ψ_0 as well as the ground state energy E_0.

2.1. Quartic anharmonic correction

Besides the vibrational mode along z-direction described using the FDM, we capture the vibrational motions along the x and y axes using a three-point fitted potential

$$V_\perp(x)=\frac{1}{2}kx^2+\gamma x^4$$

where k is the effective spring constant, γ represents the constant for the quartic correction term and a constant. The fitted parameters for the systems studied in the manuscript are presented in Suppl. Tables 15, 17 and 19.

Using the harmonic oscillator as reference, the first order perturbation theory correction to the ground state energy is

$$\Delta E_0 = \langle \Psi_0 | \gamma x^4 | \Psi_0 \rangle = \frac{3\gamma\hbar^2}{4\mu k}$$

Such that the total zero-point energy of the system is

$$E_0^{quartic} = \frac{\hbar}{2}\sqrt{\frac{k}{\mu}} + \frac{3\gamma\hbar^2}{4\mu k}$$

Similarly, the first excited state energy is

$$E_1^{quartic} = \frac{3\hbar}{2}\sqrt{\frac{k}{\mu}} + \frac{15\gamma\hbar^2}{4\mu k}$$

The corresponding energy difference was used to compute the frequencies in Table 3 in the main manuscript,

$$\Delta E_{0-1}^{quartic} = \hbar\sqrt{\frac{k}{\mu}} + \frac{3\gamma\hbar^2}{\mu k}$$

Supplementary Table 3. Vibrational analysis for Cu(I)–He complexes at the MP2/def2-TZVPP level. Harmonic and VPT2 frequencies (cm⁻¹) of adsorbed ^{4}He (in parentheses ^{3}He) for all Cu(I)-complexes. $\nu_{\text{harm}}^{\text{full}}(\nu_{\text{VPT2}}^{\text{full}})$ are derived from full normal mode analysis; whereas $\nu_{\text{harm}}^{\text{He}}(\nu_{\text{VPT2}}^{\text{He}})$ keep only the three degrees of freedom of the adsorbed He atom. $\nu_{\text{numerical}}^{\text{MCID}}$ values obtained using $V_{\text{MCID}}(z)$ potential for the stretching mode ν_s, and a quartic potential for the orthogonal, $\nu_{\perp 1}$ and $\nu_{\perp 2}$. The reduced mass used is that of Cu(I) and He ($\mu_{\text{Cu-He}}$).

Systems	*Modes*	ν_{harm}^{full}	ν_{harm}^{He} *3DOFs*[a]	ν_{VPT2}^{full}	ν_{VPT2}^{He} *3DOFs*[a]	$\nu_{numerical}^{MCID}$
Cu^+He	ν_s	187.4 (214.2)	187.4 (214.2)	134.1 (144.7)	134.1 (144.7)	140.1 (154.0)
$Cu^+(OH^-)He$	ν_s	512.4 (580.2)	509.1 (586.5)	415.5 (466.5)	413.1 (459.0)	444.2 (494.9)
	$\nu_{\perp 1}$	226.9 (251.3)	191.2 (220.2)	231.5 (250.9)	177.6 (202.1)	197.2 (225.4)
	$\nu_{\perp 2}$	230.5 (255.1)	191.4 (220.5)	235.2 (255.5)	178.4 (203.3)	199.5 (228.1)
$Cu^+(H_2O)He$	ν_s	337.9 (370.7)	359.4 (414.0)	272.3 (308.7)	277.2 (304.9)	301.2 (333.2)
	$\nu_{\perp 1}$	118.7 (131.9)	101.0 (116.3)	109.0 (97.4)	89.5 (99.8)	100.4 (114.8)
	$\nu_{\perp 2}$	122.4 (138.9)	103.8 (119.6)	138.3 (117.6)	92.5 (103.0)	107.0 (122.4)
$Cu^+(H_2O)(OH^-)He$	ν_s	456.8 (515.4)	459.8 (529.7)	345.2 (390.5)	355.7 (391.5)	384.2 (424.7)
	$\nu_{\perp 1}$	162.8 (177.6)	162.7 (187.4)	148.2 (165.9)	152.4 (173.1)	174.9 (200.0)
	$\nu_{\perp 2}$	198.3 (219.8)	166.7 (192.1)	177.7 (197.6)	149.4 (169.8)	224.2 (256.3)
$Cu^+(F^-)He$	ν_s	518.9 (577.9)	527.8 (608.5)	427.9 (490.0)	431.9 (480.7)	495.6 (554.0)
	$\nu_{\perp 1}$	215.7 (239.1)	180.1 (207.5)	199.1 (219.0)	168.8 (192.5)	185.8 (212.5)
	$\nu_{\perp 2}$	215.7 (239.1)	180.1 (207.5)	199.1 (219.0)	168.8 (192.5)	185.8 (212.5)
$Cu^+(Cl^-)He$	ν_s	456.6 (484.4)	378.3 (435.8)	420.5 (426.0)	277.7 (302.3)	317.9 (350.8)
	$\nu_{\perp 1}$	162.0 (181.2)	137.5 (158.4)	145.2 (159.8)	122.8 (138.8)	140.5 (160.6)
	$\nu_{\perp 2}$	162.0 (181.2)	137.5 (158.4)	145.2 (159.8)	122.8 (138.8)	140.5 (160.6)

$Cu^+(Br^-)He$	ν_s	374.2 (411.6)	329.4 (379.5)	300.6 (321.7)	231.4 (249.4)	271.8 (296.9)
	$\nu_{\perp 1}$	143.4 (161.2)	125.3 (144.4)	126.2 (139.1)	109.6 (123.5)	132.5 (151.5)
	$\nu_{\perp 2}$	143.4 (161.2)	125.4 (144.4)	126.2 (139.1)	109.6 (123.5)	132.5 (151.5)
$Cu^+(HS^-)He$	ν_s	312.1 (353.6)	312.0 (359.4)	215.0 (230.4)	213.0 (227.2)	238.9 (259.3)
	$\nu_{\perp 1}$	147.9 (165.3)	131.1 (151.0)	136.4 (148.1)	113.3 (127.4)	131.8 (150.7)
	$\nu_{\perp 2}$	158.3 (177.0)	138.3 (159.3)	146.3 (158.9)	121.4 (136.0)	131.8 (150.7)

Supplementary Table 4. Summary of computed zero-point energies for adsorbed He isotopes on Cu(I) complexes. Zero-point energy (ZPE in kJ mol^{-1}) of ^{4}He (in parentheses, ^{3}He) derived from the harmonic frequencies at the CCSD(T)/ aug-cc-pVTZ and DLPNO-CCSD(T)/cc-pVTZ(aug-cc-pVTZ for Cu, He) levels. 'Full-harmonics', is derived from full normal mode analysis; whereas '3DOFs-harmonics' keeps only the three degrees of freedom of He atom. 3DOFs-numericals values obtained using $V_{MCID}(z)$ potential for the stretching mode ν_s, and a quartic potential for the orthogonal, $\nu_{\perp 1}$ and $\nu_{\perp 2}$. The reduced mass used is that of Cu(I) and He ($\mu_{Cu\text{-}He}$).

	CCSD(T)/aug-cc-pVTZ				DLPNO- CCSD(T)
Systems	Analytical solutions			Numericals	Numericals
		Full, harmonic	3DOFs, harmonic	3DOFs	3DOFs
Cu^+He	$ZPE_{str.}$	1.362 (1.558)	1.362 (1.558)	1.324 (1.503)	1.354 (1.541)
$Cu^+(OH^-)He$	$ZPE_{str.}$	2.714 (3.08)	2.708 (3.120)	2.674 (3.051)	2.826 (3.209)
	$ZPE_{\perp 1}$	1.161 (1.287)	0.981 (1.130)	0.830 (0.950)	0.994 (1.137)
	$ZPE_{\perp 2}$	1.203 (1.333)	0.997 (1.148)	1.022 (1.169)	1.027 (1.174)
$Cu^+(H_2O)He$	$ZPE_{str.}$	1.988 (2.201)	2.065 (2.379)	2.224 (2.526)	2.235 (2.544)
	$ZPE_{\perp 1}$	0.639 (0.714)	0.555 (0.639)	0.602 (0.689)	0.617 (0.705)
	$ZPE_{\perp 2}$	0.678 (0.755)	0.574 (0.662)	0.605 (0.692)	0.633 (0.724)
$Cu^+(H_2O)(OH^-)He$	$ZPE_{str.}$	2.233 (2.288)	2.225 (2.564)	2.295 (2.610)	2.141 (2.473)
	$ZPE_{\perp 1}$	0.837 (0.935)	0.813 (0.938)	0.948 (1.084)	0.998 (1.142)
	$ZPE_{\perp 2}$	0.955 (1.063)	0.824 (0.948)	1.122 (1.283)	1.036 (1.184)
$Cu^+(F^-)He$	$ZPE_{str.}$	2.905 (3.258)	2.938 (3.385)	2.635 (3.037)	2.954 (3.362)
	$ZPE_{\perp 1}$	1.148 (1.271)	0.952 (1.097)	0.976 (1.116)	0.951 (1.088)
	$ZPE_{\perp 2}$	1.150 (1.272)	0.954 (1.099)	0.976 (1.116)	0.951 (1.088)
$Cu^+(Cl^-)He$	$ZPE_{str.}$	2.195 (2.344)	2.321 (2.674)	2.213 (2.519)	2.421 (2.740)

	$ZPE_{\perp 1}$	0.869 (0.972)	0.760 (0.876)	0.784 (0.896)	0.631 (0.721)
	$ZPE_{\perp 2}$	0.869 (0.972)	0.760 (0.876)	0.784 (0.896)	0.631 (0.721)
$Cu^+(Br^-)He$	$ZPE_{str.}$	2.119 (2.317)	1.887 (2.174)	1.888 (2.161)	2.110 (2.389)
	$ZPE_{\perp 1}$	0.782 (0.783)	0.706 (0.813)	0.722 (0.826)	0.768 (0.878)
	$ZPE_{\perp 2}$	0.880 (0.881)	0.706 (0.814)	0.722 (0.826)	0.768 (0.878)
$Cu^+(HS^-)He$	$ZPE_{str.}$	1.768 (2.003)	1.769 (2.038)	1.408 (1.634)	1.817 (2.058)
	$ZPE_{\perp 1}$	0.771 (0.863)	0.682 (0.785)	0.745 (0.852)	0.634 (0.725)
	$ZPE_{\perp 2}$	0.824 (0.922)	0.721 (0.831)	0.769 (0.879)	0.641 (0.733)

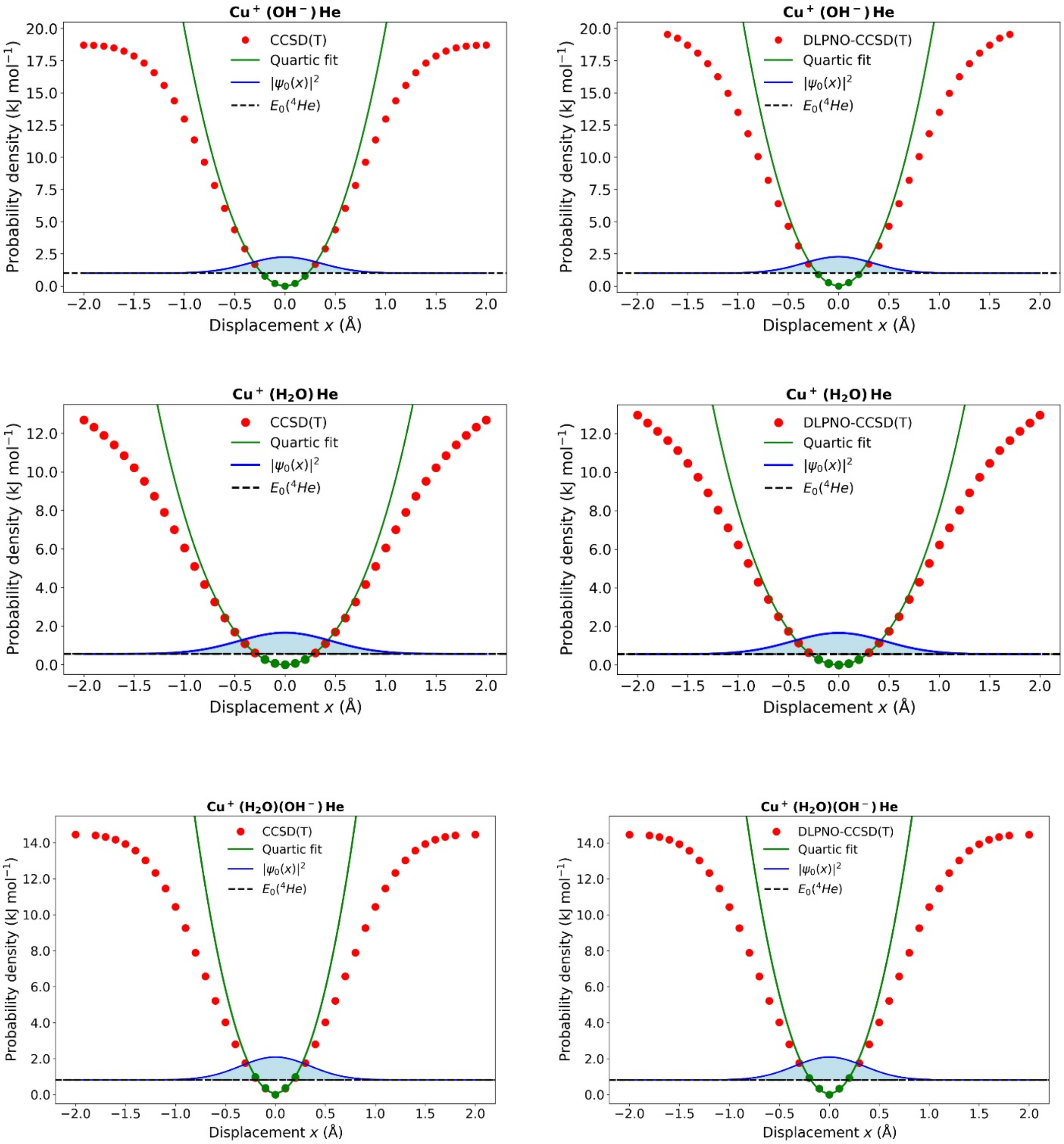

Supplementary Fig. 4. Potential energy values along x direction. All the potential curves were obtained at CCSD(T)/aug-cc-pVTZ (left side) and DLPNO-CCSD(T)/cc-pVTZ(aug-cc-pVTZ for Cu, He) (right side) levels. The red dots mark the data points and the green curve represents the quartic potential fit using three data points (green dots). The black dashed line indicates the ground state energy for ^{4}He, and the blue curve shows the ground-state probability density $|\psi_0(x)|^2$ derived from a one-dimensional Schrödinger's equation.

3. Supplementary Note 3. Detailed Calculations of adsorbed He Isotope Separation on Strongly Attractive Adsorption Site

The separation factor for adsorbed helium isotopes is defined as:

$$\alpha({}^{4}He/{}^{3}He) = \frac{K_{ad}({}^{4}He)}{K_{ad}({}^{3}He)}$$

where $K_{ad}({}^{4}He)$ and $K_{ad}({}^{3}He)$ represent the equilibrium adsorption constants for the heavier He isotope (${}^{4}He$) to the lighter one (${}^{3}He$), respectively, given by:

$$K_{ad}({}^{4}He) = \frac{q^{ad}_{{}^{4}He}}{q^{g}_{{}^{4}He}} \text{ and } K_{ad}({}^{3}He) = \frac{q^{ad}_{{}^{4}_{3}He}}{q^{g}_{{}^{3}He}}$$

with the partition functions

$$q^{ad}_{{}^{4}He} = q^{ad}_{str,{}^{4}He} \times q^{ad}_{\perp 1,{}^{4}He} \times q^{ad}_{\perp 2,{}^{4}He}.$$

$$q^{ad}_{{}^{3}He} = q^{ad}_{str,{}^{3}He} \times q^{ad}_{\perp 1,{}^{3}He} \times q^{ad}_{\perp 2,{}^{3}He}.$$

Here M denotes the metal adsorption site, that is, Cu(I) in this study.

The energy levels of a quantum harmonic oscillator are given as:

$$E(\nu) = \hbar \cdot \omega \cdot \left(\nu + \frac{1}{2}\right), \qquad \text{with } \nu = 0,1,2, \ldots, \text{ and } \omega = \sqrt{\frac{k}{\mu}},$$

where ν is the vibrational quantum number, ω is the harmonic frequency, k is the force constant from the harmonic potential and μ is the reduced mass.

The harmonic vibrational partition function for the m_{th} mode ($m = str$, $\perp_1, \perp_2$) of isotope i is expressed as follows:

$$q^{ad}_{m,i} = \sum_{\nu=0}^{\infty} exp\left(-\frac{E_{m,i}(\nu)}{RT}\right) = \frac{exp(-E_{m,i}(0)/R\cdot T)}{1-exp(-2\cdot E_{m,i}(0)/RT)}.$$

where R is the ideal gas constant, and T is the temperature and $E_{m,i}(\nu)$ is the energy of the ν_{th} vibrational state for the mode m and isotope i.

To go beyond the harmonic approximation, we used the ground state energy $E_{m,i}(0)$ obtained from FDM for the Cu-He stretch together with the quartic potential energy for the two He degrees of freedom normal to the Cu-He bond (detailed in Supplementary Note 2) to calculate the partition functions $q^{ad}_{^4He}$ and $q^{ad}_{^3He}$.

This gives the ratio of the partition functions for the adsorbed phase,

$$\frac{q^{ad}_{m,^4He}}{q^{ad}_{m,^3He}} = \left(\frac{exp\left(-E_{m,\,^4He}(0)/\mathrm{R}T\right)}{1 - exp\left(-2\,E_{m,\,^4He}(0)/\mathrm{R}T\right)} \right) \cdot \left(\frac{exp\left(-E_{m,\,^3He}(0)/\mathrm{R}T\right)}{1 - exp\left(-2\,E_{m,\,^3He}(0)/\mathrm{R}T\right)} \right)^{-1}$$

$$= \frac{exp\left(-E_{m,\,^4He}(0)/R\,T\right)}{exp\left(-E_{m,\,^3He}(0)/R\,T\right)} \cdot \frac{1 - exp\left(-2\,E_{m,\,^3He}(0)/\mathrm{R}T\right)}{1 - exp\left(-2\,E_{m,\,^4He}(0)/\mathrm{R}T\right)}$$

In the ideal gas, the translational partition function is defined as:

$$q_i^{g,tr}(\tau_x, \tau_y, \tau_z) = V \cdot \left(\frac{M_i \cdot \mathrm{R} \cdot T}{2\pi \cdot \hbar^2 \cdot N_A^2} \right)^{3/2}$$

where M_i denotes the molar mass of the gas (^{3}He or ^{4}He), $\hbar$ is the reduced Planck's constant, N_A is Avogadro's constant and V, the volume, can be obtained using the ideal gas equation:

$$V = \frac{n.\mathrm{R}.T}{\rho}$$

where n represents the molar amount and ρ represents the pressure. In the calculation of the separation factors, all quantities except the molar masses ($M_{^3He}$ and $M_{^4He}$) will cancel because they are identical, leaving only:

$$\frac{q^{g,tr}_{^3He}}{q^{g,tr}_{^4He}} = \left(\frac{M_{^3He}}{M_{^4He}} \right)^{3/2}$$

The final expression for the selectivity factor is

$$\alpha(^4He/^3He) = \frac{q^{ad}_{str,^4He}}{q^{ad}_{str,^3He}} \frac{q^{ad}_{\perp_1,^4He}}{q^{ad}_{\perp_1,^3He}} \frac{q^{ad}_{\perp_2,^4He}}{q^{ad}_{\perp_2,^3He}} \left(\frac{M_{^3He}}{M_{^4He}} \right)^{3/2}$$

Note that, for the bare Cu(I)–He complex, the rotational partition function is considered and can be written as:

$$q_i^{rot} = \frac{\mathrm{R}T}{B}, \text{ with } B = \frac{\hbar^2}{2\mu_i {z_e}^2}$$

where B is the rotational constant, and z_e the equilibrium distance. This yields the partition function of the adsorbed phase for the Cu(I)–He system, expressed as:

$$q_{^4He}^{ad} = q_{str,^4He}^{ad} \times q_{^4He}^{rot}$$
$$q_{^3He}^{ad} = q_{str,^3He}^{ad} \times q_{^3He}^{rot}$$

Supplementary Table 5. Predicted separation factors ($^4He/^3He$) for adsorbed He isotopes in all Cu(I)-complexes at low temperatures. All values are based on the CCSD(T)/aug-cc-pVTZ level (for validation purposes at DLPNO-CCSD(T)/cc-pVTZ(aug-cc-pVTZ for Cu, He) level).

Temperature (K)	$Cu^+(F^-)$	$Cu^+(OH^-)$	$Cu^+(H_2O)(OH^-)$	$Cu^+(Cl^-)$	$Cu^+(Br^-)$	$Cu^+(H_2O)$	$Cu^+(HS^-)$	Cu^+
LH2 = 20	39.5 (39.5)	31.4 (1)	25.9 (27.9)	15.8 (13.2)	11.8 (13.1)	11.5 (12.3)	9.4 (8.4)	2.5 (2.6)
25	17.4 (17.4)	14.5 (16.7)	12.4 (13.2)	8.4 (7.2)	6.6 (7.2)	6.5 (6.9)	5.5 (5.0)	2.0 (2.1)
30	10.1 (10.1)	8.7 (9.7)	7.6 (8.0)	5.5 (4.9)	4.5 (4.8)	4.4 (4.7)	3.9 (3.6)	1.8 (1.8)
35	6.8 (6.8)	6.0 (6.6)	5.4 (5.6)	4.1 (3.7)	3.4 (3.7)	3.4 (3.5)	3.0 (2.8)	1.6 (1.6)
40	5.1 (5.1)	4.6 (5.0)	4.1 (4.3)	3.2 (3.0)	2.8 (3.0)	2.8 (2.9)	2.5 (2.4)	1.5 (1.5)
45	4.1 (4.1)	3.7 (4.0)	3.4 (3.5)	2.7 (2.6)	2.4 (2.5)	2.4 (2.5)	2.2 (2.1)	1.4 (1.4)
50	3.4 (3.4)	3.1 (3.3)	2.9 (3.0)	2.4 (2.3)	2.1 (2.2)	2.1 (2.2)	1.9 (1.9)	1.3 (1.3)
55	2.9 (2.9)	2.7 (2.9)	2.5 (2.6)	2.1 (2.0)	1.9 (2.0)	1.9 (2.0)	1.8 (1.7)	1.3 (1.3)
60	2.6 (2.6)	2.4 (2.6)	2.3 (2.3)	2.0 (1.9)	1.8 (1.8)	1.8 (1.8)	1.7 (1.6)	1.2 (1.2)
65	2.4 (2.4)	2.2 (2.3)	2.1 (2.1)	1.8 (1.7)	1.7 (1.7)	1.7 (1.6)	1.6 (1.5)	1.2 (1.2)
70	2.2 (2.2)	2.0 (2.1)	1.9 (2.0)	1.7 (1.6)	1.6 (1.6)	1.6 (1.6)	1.5 (1.5)	1.2 (1.2)
75	2.0 (2.0)	1.9 (2.0)	1.8 (1.8)	1.6 (1.6)	1.5 (1.5)	1.5 (1.4)	1.4 (1.4)	1.1 (1.2)
LN2 = 77	2.0 (2.0)	1.9 (1.9)	1.7 (1.8)	1.6 (1.5)	1.5 (1.5)	1.5 (1.4)	1.4 (1.4)	1.1 (1.2)

Supplementary Table 6. Influence of threshold settings on interaction parameters. Effect of TightSCF/TightPNO and VeryTightSCF/VeryTightPNO threshold settings on the calculated bond lengths d_{CuHe} and BO adsorption energies without BSSE-corrected (E_{ads}^{B0}).

	CCSD(T)/ aug-cc-pVTZ		DLPNO-CCSD(T)/cc-pVTZ(Cu-He: aug-cc-pVTZ)			
**System**	d_{CuHe} (Å)	E_{ads}^{B0} (kJ·mol⁻¹)	d_{CuHe} (Å)		E_{ads}^{B0} (kJ·mol⁻¹)	
			Tight	Very-Tight	Tight	Very-Tight
Cu^+He	2.00	–8.2	1.98	1.97	–8.7	–9.9
$Cu^+(OH^-)He$	1.72	–18.7	1.70	1.70	–20.5	–22.7
$Cu^+(H_2O)He$	1.82	–15.6	1.80	1.80	–16.5	–16.8
$Cu^+(F^-)He$	1.70	–21.1	1.70	1.70	–23.2	–23.4
$Cu^+(Cl^-)He$	1.77	–13.8	1.80	1.80	–14.0	–14.1
$Cu^+(Br^-)He$	1.83	–11.7	1.81	1.80	–12.1	–12.8
$Cu^+(HS^-)He$	1.85	–8.8	1.87	1.90	–8.8	–9.0

Supplementary Table 7. Summary of QTAIM descriptors for adsorbed He on various material models. Local QTAIM properties calculated at the PBE0-D3(BJ)/TZ2P level of theory on DLPNO-CCSD(T)/ cc-pVTZ(aug-cc-pVTZ for Cu, He) geometries. Hirshfeld charges of cluster models of molecules and materials are given for Cu in the bare system q_{Cu}, in the systems with adsorbed helium q_{CuHe} and their differences $\Delta_{ads}q_{Cu}$, as well as for the adsorbed He atoms q_{He}, electron density ρ_{BCP} and its Laplacian $\nabla^2{}_{BCP}$ at the BCP between Cu^+ and He and the distance of the BCP to the copper z_{Cu-BCP} and helium z_{He-BCP} centers.

**Systems**	q_{Cu} (e)	q_{CuHe} (e)	$\Delta_{ads}q_{Cu}$ (e)	q_{He} (e)	ρ_{BCP} $(e \cdot r_{\mathrm{Bohr}})$	$\nabla^2{}_{BCP}$ $(e \cdot r_{\mathrm{Bohr}})$	z_{Cu-BCP} (Å)	z_{He-BCP} (Å)
**6c2**	+0.565	+0.481	−0.084	+0.110	0.052	0.334	1.03	0.78
**9c3**	+0.479	+0.395	−0.084	+0.093	0.042	0.261	1.08	0.81
**2z$^-$**	+0.473	+0.417	−0.056	+0.067	0.023	0.130	1.20	0.92
**3z$^-$**	+0.523	+0.458	−0.065	+0.072	0.025	0.146	1.18	1.02
**Cu(I)-MFU-4*l***	+0.287	+0.267	−0.020	+0.019	0.006	0.026	1.54	1.18
**Cu_3Pyr_3**	+0.271	+0.262	−0.009	+0.023	0.002	0.010	2.10	1.42
**Cu(I)Porphyrin**	+0.596	+0.583	−0.013	+0.023	0.003	0.012	1.73	1.31
**Cu(I)Cu(II)$(BTC)_3$**	+0.477	+0.451	−0.026	+0.034	0.007	0.030	1.49	1.16
**Cu(I)Zn(II)$(BTC)_3$**	+0.345	+0.327	−0.018	+0.022	0.004	0.014	1.68	1.27
**Cu(I)/UiO-66**	+0.391	+0.354	−0.037	0.053	0.014	0.071	1.31	1.02
**Cu[$(Cu_4Cl)(ttpm)_2]_2$**	+0.479	+0.406	−0.073	+0.085	0.032	0.186	1.13	0.87

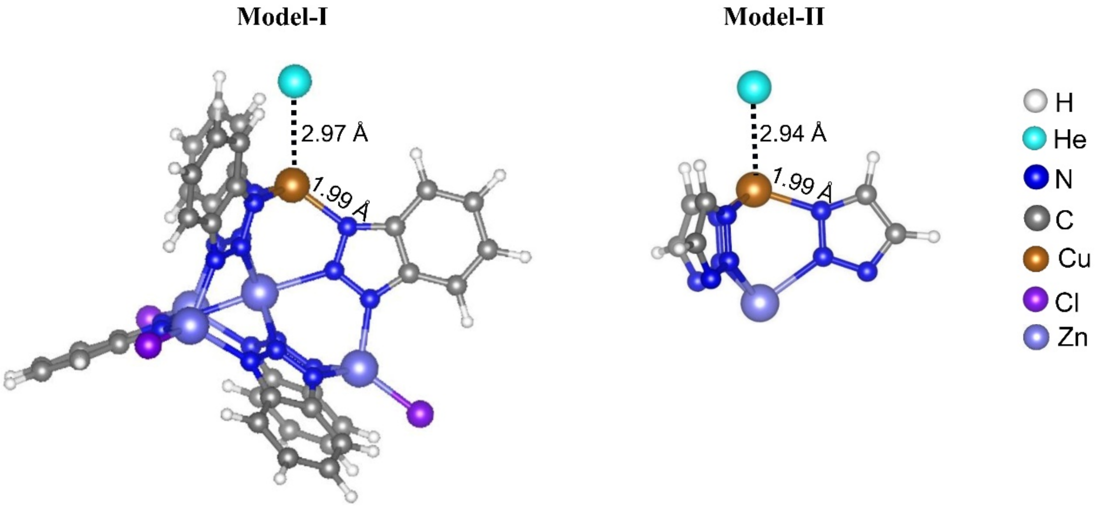

Supplementary Fig. 5. Optimized structure of the uncharged cluster containing the benzotriazolate (bta$^-$) ligand of formula Cu(I)$Zn_4Cl(bta)_6$ (Model-I) and its smaller truncated version (Model-II) at PBE0-D3/def2-TZVP level. Model-I and Model-II are used to represent the SBU of Cu(I)-MFU-4l. Selected bond distances and angles: Model-I: Cu −Zn = 3.25 Å; Cu −N (mean) = 1.99 Å; Zn −N (mean) = 1.99 Å; Cu −He (mean) =2.97 Å; N −Cu−N (mean) = 26.7° and Zn−Cu−N (mean) = 69.3°; Model-II: Cu −Zn = 3.25 Å; Cu −N (mean) = 1.99 Å; Zn −N (mean) = 2.23 Å; Cu −He (mean) = 2.94 Å; N −Cu−N (mean) = 26.7°; Zn−Cu−N (mean) = 69.3° .

Supplementary Table 8. Comparison of adsorption energies using DFT and high-level methods. Adsorption energies of adsorbed He in Model-I and Model-II using PBE0-D3/cc-pVTZ and, for validation, using DLPNO-CCSD(T)/cc-pVTZ (aug-cc-pVTZ for Cu, He) level. High-level (HL) and low-level (LL) corrections have been applied, and the estimated HL interaction energy of the large system (ΔE_{int}^{HL}) was calculated.

Levels	E_{ads}^{BO}		[a]ΔE_{int}^{HL} (kJ·mol⁻¹)
	Model-I	Model-II	HL // LL
PBE0-D3/cc-pVTZ	−2.4	−1.6	/
DLPNO-CCSD(T)/cc-pVTZ (aug-cc-pVTZ for Cu, He)	/	−1.8	−2.6

[a]ΔE_{int}^{HL}: Estimated high-level energy of the large system.
HL // LL: DLPNO-CCSD(T)/cc-pVTZ (aug-cc-pVTZ for Cu, He)// PBE0-D3//cc-pVTZ

Supplementary Table 9. Adsorbed He isotopes on various cluster models. Calculated ZPE-corrected adsorption energies (numerical method) obtained without BSSE correction and corresponding surface coverages ($\theta_{^3He}$) calculated at the DLPNO-CCSD(T)/ cc-pVTZ(aug-cc-pVTZ for Cu, He) level of theory.

Systems	E^0_{ads} *(kJ·mol⁻¹)*	$\theta_{^3He}$ (%)	
	^{3}He	T = 20 K LH2	T = 77 K LN2
6c2	–9.1	100.0	100.0
9c3	–5.7	99.9	99.8
$2z^-$	–2.4	98.5	94.5
$3z^-$	–3.1	99.3	97.2
Cu(I)-MFU-4*l*	–1.0	94.2	80.9
Cu(I)Porphyrin	–2.1	98.0	92.7
Cu(I)Cu(II)$(BTC)_3$	–1.3	95.7	85.1
Cu(I)Zn(II)$(BTC)_3$	–0.4	90.0	70.0
Cu(I)/UiO-66	– 1.9	97.6	91.3
Cu[$(Cu_4Cl)(ttpm)_2]_2$	–3.9	99.7	98.7

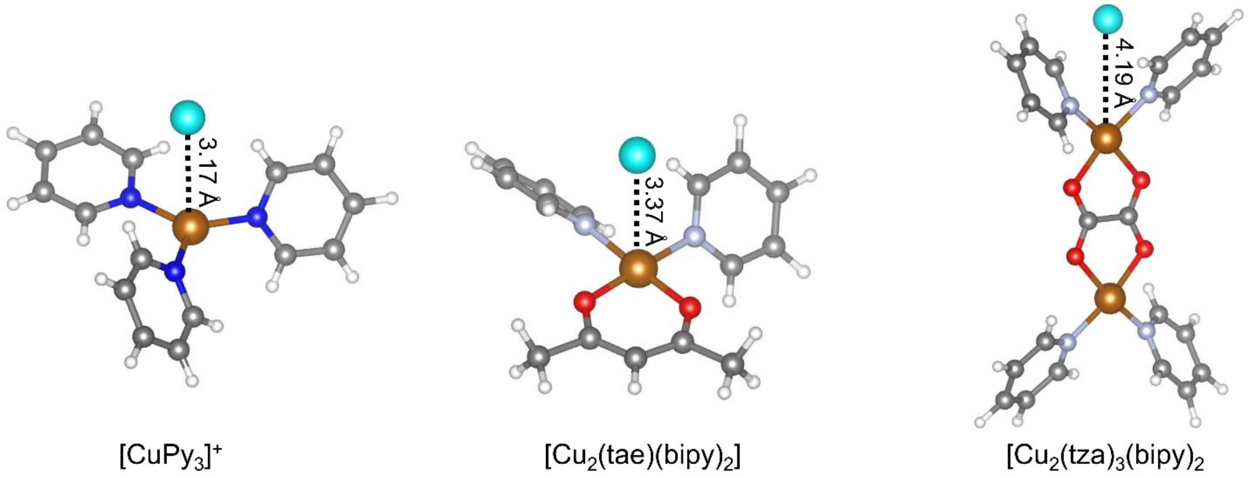

Supplementary Fig. 6. Adsorbed He atom on three SBUs from the HEALED-library. $[CuPy_3]^+$ (Py =pyridine)(13), $[Cu_2(tae)(bipy)_2]$ (bipy = bipyridine and tae = tetraacetylethane)(14) and $[Cu_2(tza)_3(bipy)_2$ (tza = tetrazole-1-acetic acid)(15) are representative structures selected. In each motif, the Cu(I)–He bond distance is highlighted.

Supplementary Table 10. Structural and energetic properties of dsorbed He isotopes on various cluster models. Bond lengths, BO adsorption energies and ZPE-corrected of He isotopes calculated at the DLPNO-CCSD(T)/cc-pVTZ(aug-cc-pVTZ for Cu, He) level of theory. Bond distances in Å and all energies in in kJ mol^{-1}.

Systems	d_{CuHe}	E_{ads}^{B0}	ZPE							
			^{4}He				^{3}He			
			$str.$	⊥$_1$	⊥$_2$	$str.+$ ⊥$_1$ + ⊥$_2$	$str.$	⊥$_1$	⊥$_2$	$str.+$ ⊥$_1$ + ⊥$_2$
6c2	1.81	−12.4	1.829	0.521	0.566	2.916	2.090	0.595	0.647	3.332
9c3	1.89	−8.4	1.545	0.408	0.420	2.373	1.754	0.466	0.481	2.701
$2z^-$	2.12	−3.3	0.681	0.019	0.095	0.795	0.773	0.022	0.109	0.904
$3z^-$	2.08	−4.7	0.805	0.298	0.322	1.425	0.915	0.341	0.368	1.624
Cu(I)-MFU-4l	2.72	−1.8	0.444	0.091	0.150	0.685	0.506	0.104	0.172	0.782
Cu(I)Porphyrin	3.04	−2.8	0.459	0.081	0.142	0.682	0.522	0.093	0.163	0.778
Cu(I)Cu(II)$(BTC)_3$	2.65	−2.6	0.437	0.306	0.372	1.115	0.515	0.350	0.425	1.290
Cu(I)Zn(II)$(BTC)_3$	2.95	−1.4	0.312	0.054	0.494	0.860	0.367	0.061	0.565	0.993
Cu(I)/UiO-66	2.33	−3.4	0.570	0.239	0.496	1.305	0.662	0.273	0.567	1.502
$Cu[(Cu_4Cl)(ttpm)_2]_2$	2.00	−5.9	1.171	0.224	0.379	1.774	1.324	0.256	0.433	2.013
Cu_3Pyr_3	3.60	−0.8	−	−	−	−	−	−	−	−

Supplementary Table 11. Predicted separation factors for adsorbed He isotopes on various cluster models of molecules and materials calculated at DLPNO-CCSD(T)/cc-pVTZ(aug-cc-pVTZ for Cu, He) level of theory.

T (K)	6c2	9c3	$Cu[(Cu_4Cl)(ttpm)_2]_2$	3*z*ˉ	Cu(I)/UiO-66	$Cu(I)Cu(II)(BTC)_3$	$Cu(I)Zn(II)(BTC)_3$	2*z*ˉ	Cu(I)porphyrin	Cu(I)-MFU-4*l*
LH2= 20	8.0	4.7	2.8	2.2	2.2	1.9	1.6	1.5	1.3	1.3
25	4.9	3.2	2.2	1.8	1.8	1.6	1.4	1.4	1.2	1.2
30	3.5	2.5	1.8	1.5	1.5	1.4	1.3	1.3	1.1	1.1
35	2.8	2.1	1.6	1.4	1.4	1.3	1.2	1.2	1.1	1.1
40	2.4	1.9	1.5	1.3	1.3	1.2	1.2	1.2	1.1	1.1
45	2.1	1.7	1.4	1.2	1.2	1.2	1.1	1.1	1.1	1.1
50	1.9	1.6	1.3	1.2	1.2	1.1	1.1	1.1	1.0	1.0
55	1.7	1.5	1.3	1.2	1.2	1.1	1.1	1.1	1.0	1.0
60	1.6	1.4	1.2	1.2	1.2	1.1	1.1	1.1	1.0	1.0
65	1.5	1.3	1.2	1.1	1.1	1.1	1.1	1.1	1.0	1.0
70	1.5	1.3	1.2	1.1	1.1	1.1	1.0	1.0	1.0	1.0
75	1.4	1.3	1.1	1.1	1.1	1.1	1.0	1.0	1.0	1.0
LN2 = 77	1.4	1.2	1.1	1.1	1.1	1.1	1.0	1.0	1.0	1.0

4. Supplementary Note 4. Vibrational mode decomposition analysis of Cu(I)/UiO-66

To understand the effect of the framework vibrations to the Cu-He binding, we have computed the vibrational modes of the Cu(I)/UiO-66 MOF cluster model at the PBE0-D3/def2SVP level of theory. From the computed IR spectrum, we have decomposed the atomic motions in terms of the displacements along a set of internal coordinates that reveals the most prominent internal coordinates involved in the framework vibrations following the approach by Teixeira and Cordeiro (*16, 17*). This vibrational mode decomposition (VMD) analysis reveals that the Cu–He stretch is highly localized, with negligible coupling to the framework vibrations. The Cu–He bond contributes significantly to only one low-frequency mode (132.3 cm^{-1}), while all other modes display minimal Cu–He involvement (<3%). This indicates that the framework motions are dynamically independent of the Cu–He vibration, and therefore, the Cu–He bond length and interaction are not significantly affected by other molecular vibrations. On the other hand, previous ab initio and IR analysis suggest that the framework phonons and skeletal vibrations are mainly localized in the 400-800 cm^{-1} region (μ_3O, Zr-O, linker bending stretching) (*18, 19*). This energetic separation indicates minimal coupling between the lattice modes and the Cu-He vibration. Thus, these results collectively suggest that the Cu–He bond length/interaction is not significantly modulated by lattice or framework vibrations.

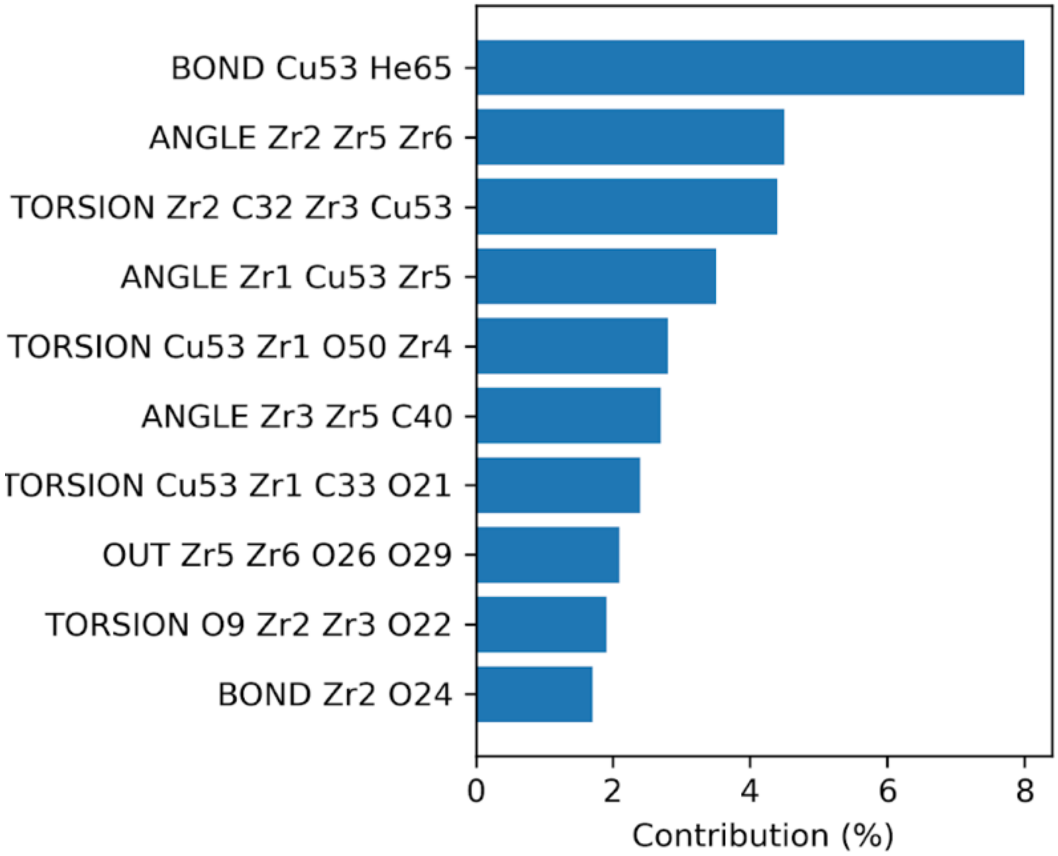

Supplementary Fig. 7. Decomposition of the vibrational mode at 132.3 cm^{-1} for the Cu(I)/UiO-66 cluster (only top ten contributions shown), highlighting the Cu–He stretch as the major contributing motion.

5. Supplementary Note 5. DVR zero-point energy comparisons

In this section we first compare the ZPEs predicted with the DVR and FDM methods for small Cu-He systems with CCSD(T)/aug-cc-pVTZ energies. Both the DVR and FDM methods describe only the three DOF of He, assuming that the rest of the atoms in the complexes are frozen. However, in the FDM calculations, we assume the three DOF are separable, such that we solve a 1D Schrödinger equation for each DOF. In contrast, in the 3D-DVR we solve a single 3D Schrödinger equation for the three DOF.

For Cu^+-He, we performed CCSD(T) calculations at different internuclear distances from 1.0 to 12.0 Å. Then we interpolated, using cubic splines, to form a DVR cubic grid from x,y,z=-3.0 to 3.0 Å with 0.2 Å separation, with the Cu atom placed at the origin. For CuOH-He, CuF-He, and Cu^+H_2O-He, CCSD(T) symmetry unique calculations were performed every 0.2 Å. Then, a tricubic interpolation scheme was used to form a DVR rectangular prism grid: from x,y=-1.0 to 1.0 Å, z=1.2 to 3.0 Å with separation of 0.1 Å. Here, the Cu atom placed at the origin, the z axis is defined by Cu-O or Cu-F axes, and the H atoms lie in the xz plane, as schematically depicted in Suppl. Fig. 8.

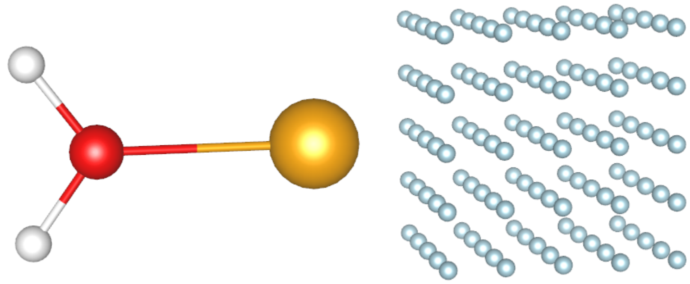

Supplementary Fig. 8. Schematic depiction of the He positions (in light blue) used to generate the Cu^+H_2O-He 3D-DVR grid.

We used the reduced mass of ^{3}He-^{63}Cu and ^{4}He-^{63}Cu in these 3D-DVR calculations. The zero point energy results are summarized in Suppl. Table 12. The 3D-DVR ^{3}He and ^{4}He are displayed in Fig. 1 panel c in the main manuscript.

Supplementary Table 12. Comparison between 3D-DVR and FDM plus quartic potential zero-point energies (in kJ/mol)

	ZPE ^4He		ZPE ^3He		ΔZPE ^3He-^4He	
X-He	DVR	FDM	DVR	FDM	DVR	FDM
Cu^+	1.328	1.324	1.507	1.503	0.180	0.179
CuF	4.523	4.587	5.146	5.269	0.623	0.682
CuOH	4.579	4.526	5.191	5.170	0.612	0.644
Cu^+H_2O	3.327	3.431	3.769	3.907	0.442	0.476

The largest DVR/FDM difference between ZPEs is 0.14 kJ/mol, which reduces to just 0.06 kJ/mol when we contrast the ΔZPE results. This difference has a minor impact of the prediction of the isotope separation factors. For these strongly bound systems, the main contribution to this factor arises from an exp(-ΔZPE/RT) term. As the FDM ΔZPEs are slightly larger than the 3D-DVR ones, employing the former ZPEs reduces the predicted separation factor. When we use the DVR values for the CuF-He system, at 20K the separation factor decreases from 39.5 to 28.0, and at 77 K from 2.0 to 1.8. However, this change in the numerical values does not affect the significance of the conclusions drawn in the manuscript.

5.1. DVR assessment of the separability of the He modes

In this section we assess the impact of the separability assumption between the Cu-He stretch mode and the two perpendicular He modes. For this, we have performed 1D- and 2D-DVR calculations for CuOH-He, CuF-He, and Cu^+H_2O-He, using the same coordinate system as in the previous section. In that coordinate system, the Cu-He stretch mode corresponds to the displacement along the z-axis, keeping the x and y He coordinates set to zero. We sampled the z-axis for each system to generate a 1D potential and then solved the 1D Schrödinger equation with DVR. Similarly, the perpendicular modes correspond to moves along the x or y axes, keeping the value of z of the optimal He position. We sampled both axes simultaneously and solved the 2D Schrödinger equation with DVR for the corresponding 2D potential. We used the same CCSD(T) energies, limits and number of points per dimension as in the 3D-DVR calculations of the previous section.

Suppl. Table 13 summarizes the results and includes a comparison with the FDM plus quartic potential results.

Supplementary Table 13. Comparison between 1D-DVR and FDM ZPEs for the Cu-He stretch and between 2D-DVR and quartic potential ZPEs for the two perpendicular He modes. The total ZPEs are also displayed. Energies in kJ/mol.

	Cu-He stretch mode		He perpendicular modes		Total	
	1D(z)-DVR	FDM	2D(xy)-DVR	quartic	1D-DVR +2D-DVR	FDM + quartic
CuF-He						
ZPE ^{4}He	2.706	2.635	1.919	1.952	4.625	4.587
ZPE ^{3}He	3.086	3.037	2.193	2.232	5.280	5.269
ΔZPE	0.380	0.402	0.274	0.280	0.655	0.682
CuOH-He						
ZPE ^{4}He	2.699	2.674	2.009	1.852	4.708	4.526
ZPE ^{3}He	3.064	3.051	2.292	2.119	5.357	5.170
ΔZPE	0.365	0.377	0.283	0.267	0.648	0.644
Cu^+H_2O-He						
ZPE ^{4}He	2.277	2.224	1.196	1.207	3.474	3.431
ZPE ^{3}He	2.585	2.526	1.368	1.381	3.953	3.907
ΔZPE	0.307	0.302	0.172	0.174	0.479	0.476

Observe that the 1D+2D-DVR ΔZPE results are in better agreement with the FDM+quartic values than the 3D-DVR of the previous section, as the largest ΔZPE difference is only 0.03 kJ/mol. Therefore, uncoupling of the He vibrational modes is the main responsible for the slight overestimation of the ZPE values discussed in the previous section.

5.2. Cu-He Stretch partition function validation

In this section we compare the approximate form of the vibrational partition function employed in the manuscript with the results of the full partition function obtained from DVR calculations. For this validation we selected the systems with the highest binding energy among the small clusters and the materials models, CuF-He and $Cu[(Cu_4Cl)(ttmp)_2]_2$-He. We constructed a 1D grid, from 1.0 to 12.0 Å with 0.1 Å separation, employing the 1D Morse plus charge induced dipole potentials for these two complexes. In these grids, we solved the 1D Schrödinger equation with DVR, using the reduced mass of ^{3}He-^{63}Cu and ^{4}He-^{63}Cu.

The full vibrational partition function is obtained as a sum over the 1D-DVR bound state energies (E_v),

$$q_{full} = \sum_{v=0} exp\,(-E_v/k_b\,T).$$

For Cu[(Cu$_4$Cl)(ttmp)$_2$]$_2$ the sum in q_{full} includes eight ^{4}He and seven ^{3}He states. For CuF q_{full} includes nine ^{4}He and eight ^{3}He states. The energies of these states are depicted in the following Figure.

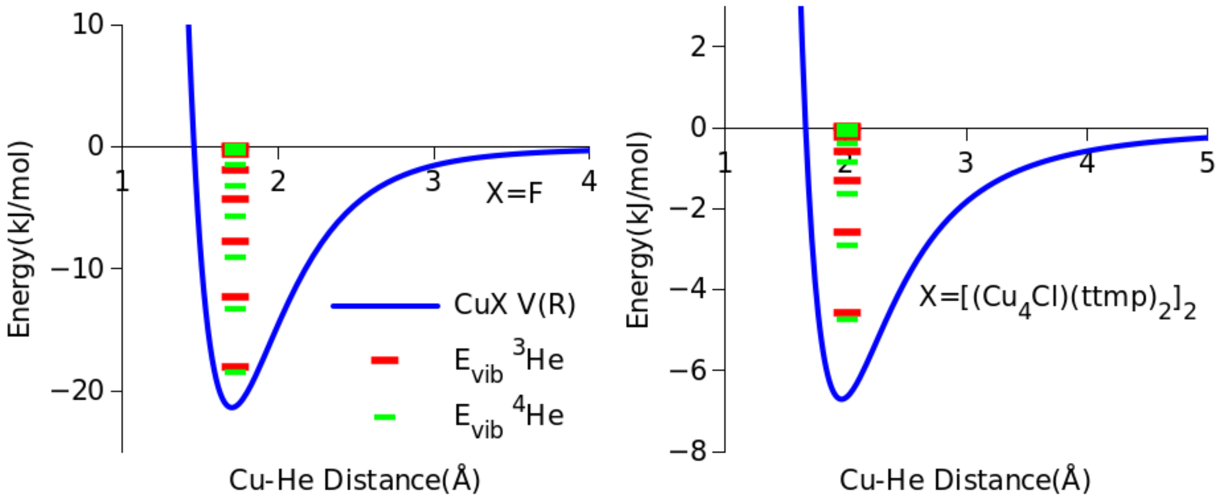

Supplementary Fig. 9. Morse plus charge induced potential energies, V(R), and 1D-DVR energies, E_{vib}, for CuF-$^{3/4}$He (left) and Cu[(Cu$_4$Cl)(ttmp)$_2$]$_2$ -$^{3/4}$He (right).

We compare the results with an approximate partition function that only considers the energy of the vibrational ground state, E_0, assuming equal separation between excited states (as is obtained for the harmonic oscillator),

$$q_{\text{approx}} = \frac{exp(-E_0/k_bT)}{1-exp(-2E_0/k_bT)}.$$

In this comparison, E_0 is also taken from the 1D-DVR calculations.

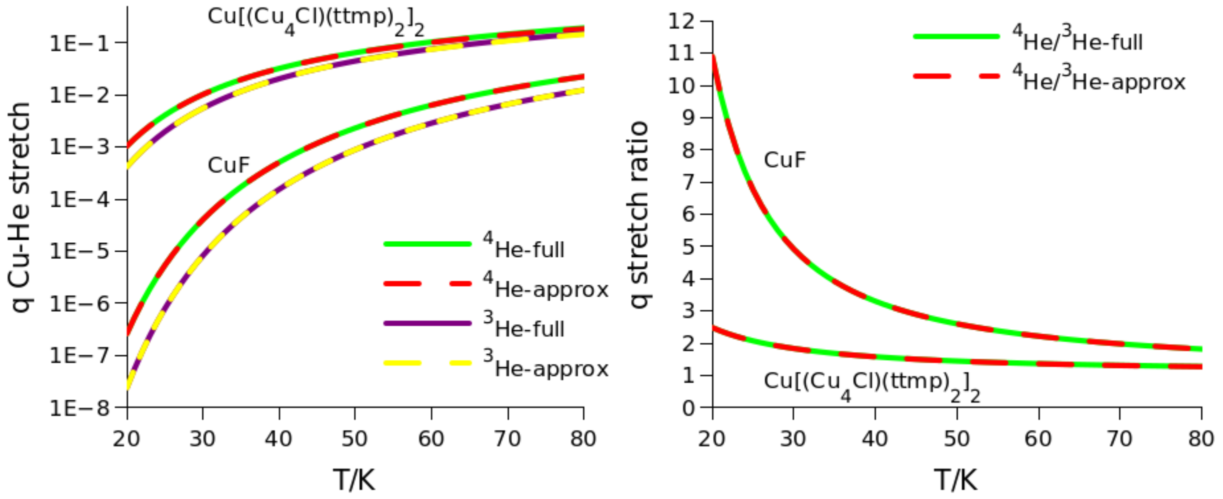

Supplementary Fig. 10. 1D-DVR partition function analysis of $^{3/4}He$ in CuF system and $Cu[(Cu_4Cl)(ttmp)_2]_2$ cluster. Left: Comparison of the 1D-DVR partition function obtained as a sum of the state energies (q_{full}) or using only the ground state energy (q_{approx}) for CuF-$^{3/4}He$ and $Cu[(Cu_4Cl)(ttmp)_2]_2$ -$^{3/4}He$. Right: Comparison of the ratio of the 1D-DVR $^{3/4}He$ partition functions for the same systems

As observed in Supp. Fig. 10, there is excellent agreement between the full and approximate forms of the vibrational partition function in the temperature range presented. The full and approximate partition functions have near perfect agreement at 20K, and their difference slightly increased as T increases. At 77 K, the error of the approximate partition function is -3.6% and -4.0%, for $^4He/^3He$ -$Cu[(Cu_4Cl)(ttmp)_2]_2$ respectively. However, when we compute the ratio of the partition function, the error reduces to only -0.4%. Over the 20-77 K range, for $^4He/^3He$ -CuF the errors in the partition functions and their ratio are always lower than 0.01%. As the two complexes mentioned here are extremes in He binding energies and $^4He/^3He$ selectivities, we can safely conclude that the partition function choice does not impact the separation factors reported in the manuscript.

5.3. He perpendicular modes partition function validation

Here we want to analyze the performance of the perturbation theory treatment we employed to describe the anharmonicity of the He vibrational modes perpendicular to the Cu-He stretch axis. We employed a quartic potential fit

$$V(x) = \frac{k}{2}x^2 + \gamma x^4,$$

where for some of our systems, the quartic coefficient γ has a negative sign. In these cases, the quartic potential only has physical meaning until the inflection point (where $4\gamma x_{infl}^3 + kx_{infl} = 0$).

As an example, we analyze the 6c2 model, where k= 11.954 kJ/mol/Å^2 and γ=-2.637 kJ/mol/Å^{4}. We constructed a 1D grid for this potential, between the inflection points -1.065 and 1.065 Å with 0.02 Å separation. In this grid, we solved the 1D Schrödinger equation with DVR, using the reduced mass of ^{3}He-^{63}Cu and ^{4}He-^{63}Cu.

The 1D-DVR calculations show three bound states, depicted in Supp. Fig. 11, for both ^{3}He and ^{4}He. The energies of these three states were employed to compute the full vibrational partition function,

$$q_{full} = \sum_{v=0} exp\,(-E_v/k_b\,T)$$

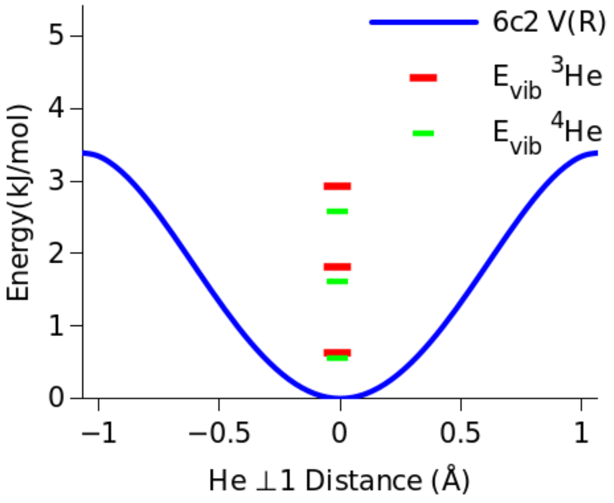

Supplementary Fig. 11. 1D-DVR bound vibrational states for the quartic potential of the He-6c2 complex.

For the quartic potential using the harmonic oscillator as reference, the first order perturbation theory correction to the ground state energy is $\Delta E_0 = \langle \Psi_0 | \gamma x^4 | \Psi_0 \rangle = \frac{3\gamma\hbar^2}{4\mu k}$, such that the total zero point energy of the system is approximated as,

$$E_{PT} = \frac{\hbar}{2}\sqrt{\frac{k}{\mu}} + \frac{3\gamma\hbar^2}{4\mu k}.$$

For 6c2, the 1D-DVR ground state energies (0.622/0.547 kJ/mol for $^3He/^4He$) and the perturbation theory results (0.624/0.548 kJ/mol for $^3He/^4He$) are within 0.02 kJ/mol. When we subtract from these energies the harmonic oscillator results (0.647/0.566 kJ/mol for $^3He/^4He$), we observe that the perturbation theory formula recovers 92% of the 1D-DVR anharmonicity energy.

To further validate our approximations, we compare the DVR partition function with the approximate partition function only considering the perturbation theory ground state energy,

$$q_{\text{approx}} = \frac{exp(-E_{PT}/k_b T)}{1 - exp(-2E_{PT}/k_b T)}.$$

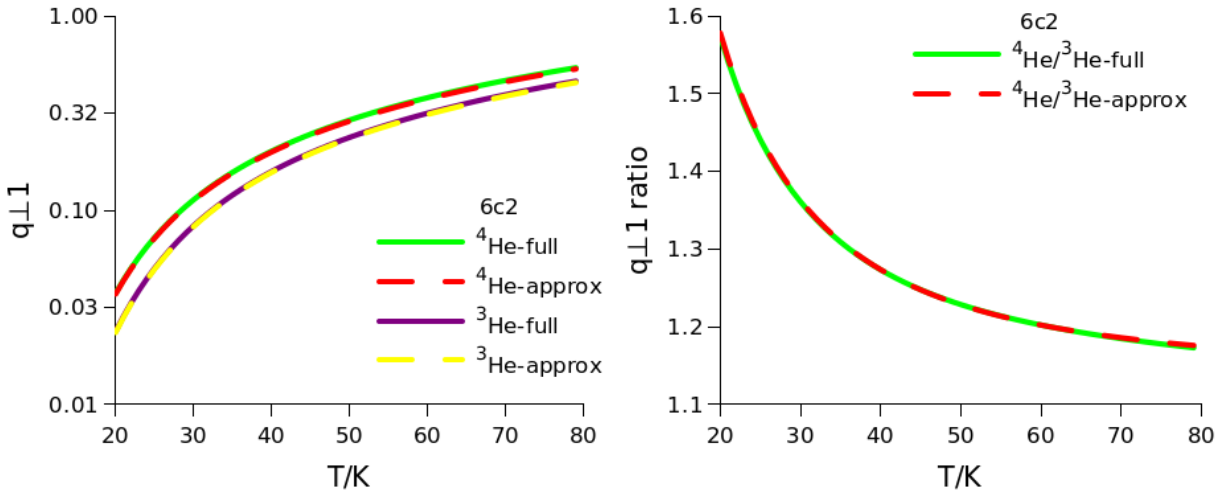

Supplementary Fig. 12. 1D-DVR partition function analysis of $^{3/4}He$ in 6c2 cluster: Comparison of q_{full} and q_{approx} partition functions and their ratios. Left: Comparison of the 1D-DVR partition function obtained as a sum of the states energies (q_{full}) or using only the ground state perturbation energy correction (q_{approx}) for 6c2-$^{3/4}He$. Right: Comparison of the ratio of the $^{3/4}He$ partition functions.

As observed in Suppl. Fig. 12., there is excellent agreement between the full and approximate forms of the vibrational partition function in the temperature range presented, with a maximum error at 77K of 1.74%. As observed in the previous section, when we compute the ratio of the partition

function, the error reduces to only -0.28%. Therefore, we can safely conclude that the perturbation theory treatment we used to account for the anharmonicity in the He perpendicular modes doesn't introduce a significant error in the prediction of the separability factors.

6. Fitted potential parameters

6.1. Small complexes

Supplementary Table 14. Morse and charge-induced dipole potential parameters with MP2 method. Fitted Morse and charge-induced dipole potential parameters describing the stretching mode potential energy curves of Cu(I) complexes computed at the MP2/def2-TZVPP level of theory without BSSE correction.

Systems	De (kJ mol^{-1})	a (Å^{-1})	ze (Å)	c (Å^{4}kJ mol^{-1})	zc (Å)	kBT (kJ mol^{-1})
Cu^+He	1.475	2.470	2.273	194.406	3.108	2.464
$Cu^+(OH^-)He$	14.449	3.058	1.712	115.510	2.493	11.735
$Cu^+(H_2O)He$	3.861	2.959	1.939	229.039	2.663	7.423
$Cu^+(H_2O)(OH^-)He$	10.442	3.246	1.730	93.089	2.528	10.386
$Cu^+(F^-)He$	19.780	3.013	1.672	92.201	2.445	10.127
$Cu^+(Cl^-)He$	8.247	3.031	1.790	86.207	2.445	10.127
$Cu^+(Br^-)He$	3.592	3.324	1.888	152.487	2.660	5.931
$Cu^+(HS^-)He$	2.701	3.418	1.915	138.751	2.708	5.892

Supplementary Table 15. Quartic potential parameters for orthogonal modes for small complexes. Parameters of quartic potential energy curves for the orthogonal modes of Cu(I) complexes, computed at the MP2/def2-TZVPP level of theory.

Systems	Potential	k ($kJ\cdot mol^{-1}\cdot Å^{-2}$)	γ ($kJ\cdot mol^{-1}\cdot Å^{-4}$)
$Cu^{+}(F^{-})He$	$\nu\perp1=\nu\perp2$	46.099	-2.407
$Cu^{+}(OH^{-})He$	$\nu\perp1$	51.897	-3.501
	$\nu\perp2$	53.123	-9.627
$Cu^{+}(H_2O)He$	$\nu\perp1$	13.469	6.564
	$\nu\perp2$	15.298	-8.752
$Cu^{+}(H_2O)(OH^{-})He$	$\nu\perp1$	40.832	-10.266
	$\nu\perp2$	67.086	-17.538
$Cu^{+}(Cl^{-})He$	$\nu\perp1=\nu\perp2$	26.348	6.419
$Cu^{+}(Br^{-})He$	$\nu\perp1=\nu\perp2$	23.437	-3.501
$Cu^{+}(HS^{-})He$	$\nu\perp1$	23.205	2.844
	$\nu\perp2$	23.194	2.957

Supplementary Table 16. Morse and charge-induced dipole potential parameters with CCSD(T) and DLPNO-CCSD(T) methods for small complexes. Fitted Morse and charge-induced dipole potential parameters describing the stretching mode potential energy curves of Cu(I) complexes computed at the CCSD(T)/aug-cc-pVTZ and DLPNO-CCSD(T)/ cc-pVTZ(aug-cc-pVTZ for Cu, He level of theories (values given in the square brackets). All without BSSE correction (see Suppl. Fig. 1).

Systems	De (kJ mol^{-1})	a (Å^{-1})	ze (Å)	c (Å^{4}kJ mol^{-1})	zc (Å)	kBT (kJ mol^{-1})
Cu^+He	2.409<br>[3.332]	2.366<br>[2.019]	2.206<br>[2.160]	273.188<br>[242.188]	3.002<br>[3.011]	2.328<br>[2.328]
$Cu^+(OH^-)He$	12.550<br>[13.140]	2.968<br>[2.870]	1.772<br>[1.773]	122.215<br>[142.131]	2.562<br>[2.565]	10.307<br>[10.307]
$Cu^+(H_2O)He$	4.557<br>[4.913]	2.797<br>[2.734]	1.979<br>[1.979]	289.617<br>[298.301]	2.693<br>[2.691]	10.309<br>[10.309
$Cu^+(H_2O)(OH^-)He$	8.445<br>[7.819]	2.983<br>[3.051]	1.818<br>[1.821]	111.344<br>[66.080]	2.636<br>[2.625]	8.496<br>[2.979]
$Cu^+(F^-)He$	14.809<br>[20.237]	2.934<br>[2.794]	1.751<br>[1.712]	120.845<br>[48.104]	2.526<br>[2.549]	10.657<br>[10.657]
$Cu^+(Cl^-)He$	7.514<br>[8.195]	2.918<br>[2.938]	1.858<br>[1.844]	144.005<br>[135.261]	2.660<br>[2.643]	8.450<br>[8.450]
$Cu^+(Br^-)He$	4.089<br>[4.856]	2.962<br>[2.922]	1.947<br>[1.926]	197.543<br>[186.690]	2.714<br>[2.700]	8.318<br>[8.318]
$Cu^+(HS^-)He$	3.949<br>[5.858]	2.953<br>[2.602]	1.946<br>[1.961]	143.078<br>[99.545]	2.526<br>[2.820]	10.657<br>[6.978]

Supplementary Table 17. Quartic potential parameters for orthogonal modes for small complexes. Parameters of quartic potential energy curves for the orthogonal modes of Cu(I) complexes, computed at the CCSD(T)/aug-cc-pVTZ and DLPNO-CCSD(T)/ cc-pVTZ(aug-cc-pVTZ for Cu, He) level of theory (values in parenthesis).

Systems	Potential	k (kJ·mol^{-1}· Å^{-2})	γ (kJ·mol^{-1}· Å^{-4})
$Cu^{+}(F^{-})He$	$\nu_{\perp 1} = \nu_{\perp 2}$	35.580 (33.377)	1.094 (4.668)
$Cu^{+}(OH^{-})He$	$\nu_{\perp 1}$	25.739 (36.870)	5.802 (9.275)
	$\nu_{\perp 2}$	39.015 (39.330)	-9.622 (-11.232)
$Cu^{+}(H_2O)He$	$\nu_{\perp 1}$	13.534 (14.188)	0.438 (-0.419)
	$\nu_{\perp 2}$	13.670 (14.974)	-6.126 (-3.743)
$Cu^{+}(H_2O)(OH^{-})He$	$\nu_{\perp 1}$	33.525 (37.200)	-8.480 (-10.630)
	$\nu_{\perp 2}$	46.983 (40.045)	-1.238 (-1.167)
$Cu^{+}(Cl^{-})He$	$\nu_{\perp 1} = \nu_{\perp 2}$	22.925 (14.842)	-1.532 (3.087)
$Cu^{+}(Br^{-})He$	$\nu_{\perp 1} = \nu_{\perp 2}$	19.455 (21.999)	1.3127 (-6.486)
$Cu^{+}(HS^{-})He$	$\nu_{\perp 1}$	20.720 (14.988)	-4.157 (3.640)
	$\nu_{\perp 2}$	22.054 (15.320)	-6.524 (3.657)

6.2. Cluster models of molecules and materials

Supplementary Table 18. Morse and charge-induced dipole potential parameters for cluster models of molecules and materials. Fitted Morse and charge-induced dipole potential parameters describing the stretching mode potential energy curves of Cu(I) complexes computed at DLPNO-CCSD(T)/ cc-pVTZ(aug-cc-pVTZ for Cu, He level of theory.

Systems	De (kJ mol^{-1})	a ($Å^{-1}$)	ze (Å)	c ($Å^4$kJ mol^{-1})	zc (Å)	kBT (kJ mol^{-1})
6c2	3.027	2.853	2.025	305.219	2.777	2.545
9c3	1.086	3.074	2.129	277.010	2.835	2.275
$2z^-$	0.608	1.897	2.525	156.660	2.835	3.969
$3z^-$	1.000	1.853	2.495	226.226	3.180	3.382
Cu(I)-MFU-4*l*	0.862	1.673	2.910	118.967	3.090	1.649
Cu(I)Porphyrin	1.097	1.733	3.249	549.867	4.530	1.593
$Cu(I)Cu(II)(BTC)_3$	1.614	1.733	2.710	143.567	3.915	1.752
$Cu(I)Zn(II)(BTC)_3$	0.723	1.600	3.028	176.476	4.110	1.267
Cu(I)/UiO-66	2.276	1.723	2.297	70.746	3.542	3.244
$Cu[(Cu_4Cl)(ttpm)_2]_2$	0.314	2.695	2.456	268.643	3.000	4.156

Supplementary Table 19. Quartic potential parameters for orthogonal modes for cluster models of molecules and materials. Parameters of quartic potential energy curves for the orthogonal modes of Cu(I) complexes, computed at the DLPNO-CCSD(T)/ cc-pVTZ(aug-cc-pVTZ for Cu, He) level of theory.

Systems	Potential	k (kJ·mol^{-1}· Å^{-2})	**γ** (kJ·mol^{-1}· Å^{-4})
6c2	$\nu_{\perp 1}$	10.121	2.359
	$\nu_{\perp 2}$	11.954	-2.637
9c3	$\nu_{\perp 1}$	6.207	3.814
	$\nu_{\perp 2}$	6.578	1.603
2z$^-$	$\nu_{\perp 1}$	0.014	-0.002
	$\nu_{\perp 2}$	0.337	-0.083
3z$^-$	$\nu_{\perp 1}$	3.323	0.781
	$\nu_{\perp 2}$	3.865	-0.508
Cu(I)-MFU-4*l*	$\nu_{\perp 1}$	0.307	0.029
	$\nu_{\perp 2}$	0.845	-0.182
Cu(I)Porphyrin	$\nu_{\perp 1}$	0.245	0.066
	$\nu_{\perp 2}$	0.757	0.431
Cu(I)Cu(II)(BTC)$_3$	$\nu_{\perp 1}$	3.491	-0.814
	$\nu_{\perp 2}$	5.153	1.177
Cu(I)Zn(II)(BTC)$_3$	$\nu_{\perp 1}$	0.107	0.039
	$\nu_{\perp 2}$	9.096	-2.820
Cu(I)/UiO-66	$\nu_{\perp 1}$	2.127	0.438
	$\nu_{\perp 2}$	9.185	-3.289
Cu[(Cu$_4$Cl)(ttpm)$_2$]$_2$	$\nu_{\perp 1}$	1.874	0.650
	$\nu_{\perp 2}$	5.356	0.597

Supplementary references

1. *Gaussian 16 (Revision C.01)* (Wallingford CT, 2016., 2016).
2. F. Neese, Software update: The ORCA program system—Version 5.0. *WIREs Comput Mol Sci* **12** (2022).
3. *Theor. Chim. Acta* (1977).
4. K. Devi, S. M. N. V. T. Gorantla, K. C. Mondal, EDA-NOCV analysis of carbene-borylene bonded dinitrogen complexes for deeper bonding insight: A fair comparison with a metal-dinitrogen system. *Journal of computational chemistry* **43**, 757–777 (2022).
5. M. Mitoraj, A. Michalak, Donor–Acceptor Properties of Ligands from the Natural Orbitals for Chemical Valence. *Organometallics* **26**, 6576–6580 (2007).
6. M. Mitoraj, A. Michalak, Applications of natural orbitals for chemical valence in a description of bonding in conjugated molecules. *J Mol Model* **14**, 681–687 (2008).
7. M. P. Mitoraj, A. Michalak, T. Ziegler, A Combined Charge and Energy Decomposition Scheme for Bond Analysis. *Journal of chemical theory and computation* **5**, 962–975 (2009).
8. E.J. Baerends, N.F. Aguirre, N.D. Austin, J. Autschbach, F.M. Bickelhaupt, R. Bulo, C. Cappelli, A.C.T. van Duin, F. Egidi, C. Fonseca Guerra, A. Förster, M. Franchini, T.P.M. Goumans, T. Heine, M. Hellström, C.R. Jacob, L. Jensen, M.V. Krykunov, E. van Lenthe, A. Michalak, M. Mitoraj, J. Neugebauer, V.P. Nicu, P.H.T. Philipsen, H. Ramanantoanina, R. Rüger, G. Schreckenbach, M. Stener, M. Swart, J.M. Thijssen, T. Trnka, L. Visscher, A.L. Yakovlev, S.J.A. van Gisbergen, *The Amsterdam Modeling Suite (AMS), version 2025.105* (SCM – Theoretical Chemistry, Vrije Universiteit Amsterdam, The Netherlands, 2025).
9. E. van Lenthe, E. J. Baerends, Optimized Slater-type basis sets for the elements 1-118. *Journal of computational chemistry* **24**, 1142–1156 (2003).
10. *Comment on "Observation of alkaline earth complexes M(CO)8 (M = Ca, Sr, or Ba) that mimic transition metals"* (2019).
11. G. Deng, S. Pan, G. Wang, L. Zhao, M. Zhou, G. Frenking, Side-On Bonded Beryllium Dinitrogen Complexes. *Angewandte Chemie International Edition* **59**, 10603–10609 (2020).
12. S. Mazumder, *Numerical methods for partial differential equations*, *Finite difference and finite volume methods* (Academic Press, Amsterdam, 2016).

13. C.-Z. Xie, Z.-F. Zhang, B.-F. Zhang, X.-Q. Wang, R.-J. Wang, G.-Q. Shen, D.-Z. Shen, B. Ding, A Novel 3D Cu I Metal–Organic Framework with Middle-Size Channels Despite the Sixfold ThSi 2 Interpenetrating Topological Structure. *Eur J Inorg Chem* **2006**, 1337–1340 (2006).
14. B. S. Luisi, V. C. Kravtsov, B. D. Moulton, An (8,3)-a 3D Coordination Network and Concomitant Three-Connected Supramolecular Isomers. *Crystal Growth & Design* **6**, 2207–2209 (2006).
15. X. He, H.-M. Guo, Z.-X. Wang, M. Shao, Syntheses, structures and properties of two copper coordination polymers constructed by tetrazole-1-acetic acid as starting material. *Inorganic Chemistry Communications* **47**, 9–12 (2014).
16. F. Teixeira, *vibAnalysis*, *GitHub repository* (2025).
17. F. Teixeira, M. N. D. S. Cordeiro, Improving Vibrational Mode Interpretation Using Bayesian Regression. *Journal of chemical theory and computation* **15**, 456–470 (2019).
18. L. Valenzano, B. Civalleri, S. Chavan, S. Bordiga, M. H. Nilsen, S. Jakobsen, K. P. Lillerud, C. Lamberti, Disclosing the Complex Structure of UiO-66 Metal Organic Framework: A Synergic Combination of Experiment and Theory. *Chem. Mater.* **23**, 1700–1718 (2011).
19. K. I. Hadjiivanov, D. A. Panayotov, M. Y. Mihaylov, E. Z. Ivanova, K. K. Chakarova, S. M. Andonova, N. L. Drenchev, Power of Infrared and Raman Spectroscopies to Characterize Metal-Organic Frameworks and Investigate Their Interaction with Guest Molecules. *Chemical Reviews* **121**, 1286–1424 (2021).
